# The power of the family in times of pandemic: Cross-country evidence from 93 countries

**DOI:** 10.1016/j.ssmph.2024.101698

**Published:** 2024-07-04

**Authors:** Ming Gu

**Affiliations:** a Global Health Research Center, Duke Kunshan University, No. 8 Duke Avenue, Kunshan, Jiangsu, 215316, China; b Division of Social Sciences, Duke Kunshan University, No. 8 Duke Avenue, Kunshan, Jiangsu, 215316, China

**Keywords:** Family ties, Culture, COVID-19, Social distancing, Vaccination

## Abstract

The majority of the hospitalizations and deaths associated with COVID-19 occurred in people over the age of 65. In addition, previous studies have shown that intergenerational contacts played a key role in COVID-19-related infection and fatality. This paper utilized two large-scale multinational surveys to uncover the important role of family ties in infection prevention across 93 countries. Using the World Values Survey, we measured country-level family ties emphasizing respondents’ view of their parents. We elicited individual willingness to uptake infection prevention measures from a panel study conducted each month in the early phase of the COVID-19 pandemic between March 2020 and July 2021. We find that in countries with stronger family ties, people show more support for non-pharmaceutical interventions and higher vaccine acceptance; moreover, young people are more supportive of mandatory vaccination. The association between strength of family ties and compliance with infection prevention measures was salient before COVID-19 vaccines became available and was persistent before the global vaccination coverage reached 25%.

## Highlights

• Stronger family ties are associated with more willingness to uptake infection prevention measures.
• Young adults show more support for mandatory vaccination in countries with stronger family ties.
• The significance of this association persisted until the global vaccination coverage reached 25%.
• Cultural values can be incorporated in policy tools in resolving future public health crises.

## 1 Introduction

By the time the World Health Organization declared an end to COVID-19 as a global health emergency in May 2023, COVID-19 had infected more than 765 million people and contributed to nearly seven million deaths worldwide (News, 2023, 5 May 2023). Reflecting on this calamity, which factors may have affected people's willingness to partake infection prevention practices during the onset of a global pandemic piqued the interests of scholars and policymakers. To answer this question, a large body of social sciences literature has endeavored to examine the effects of cultural variation on public health compliance attitudes and behaviors (Algan et al., 2021; Backhaus et al., 2023; Bargain & Aminjonov, 2020; Barrios et al., 2021; Bicchieri et al., 2021; Chen et al., 2021; Deopa & Fortunato, 2021; Leonhardt & Pezzuti, 2021; Lin et al., 2021; Merkley & Loewen, 2021; Roswag et al., 2024; Sturgis et al., 2021) and to find non-monetary incentives to boost vaccine uptake (Chang et al., 2023; Dai et al., 2021; Rabb et al., 2022).

Transmission among family members was a key challenge during the COVID-19 pandemic. Age was a risk factor affecting the severity and outcome of COVID-19 patients, and COVID-19 infection was particularly dangerous for the elderly (Mueller et al., 2020). Furthermore, anecdotal evidence in news articles and academic studies utilizing mobility data have shown that younger adults were responsible for a large percentage of the spread (Monod et al., 2021), and intergenerational contacts were a key factor in the infection spread among the elderly (Dowd et al., 2020; Esteve et al., 2020; Mazzonna & Gatti, 2023). Understanding the factors that influence transmission within households is an important component of infection control.

This study aims to examine the role of family ties plays in individual willingness to uptake infection prevention measures. Alesina and Giuliano (2010) demonstrated the importance of family ties by documenting its impact on a number of socioeconomic outcomes, including living arrangements, geographical mobility, household division of roles inside the household and female and youth labor force participation. In our study, we hypothesize that people would have higher willingness to take precautionary measures to limit the spread of the virus in countries where family ties were stronger. And we adopted the methodology presented by Alesina and Giuliano (2010) to measure the strength of family ties using the World Values Survey (WVS) (Inglehart et al., 2022). It is worth noting that the original measure of family ties by Alesina and Giuliano (2010) did not have a directional emphasis, that is, both children's love and respect for parents and parental duties towards children were equally important in constructing their measure. However, given that COVID-19 virus causes more dire consequences among the elderly, one innovation of our study is adapting this measure by putting emphasis on how children value their parents, which has some resemblance to the idea of filial piety.

We examine the relationship between willingness to uptake infection control measures and cultural norms with respect to family relationships by combining two large-scale multinational surveys. We used measures of individual-level willingness to uptake infection prevention behaviors from PsyCorona survey (Agostini et al., 2022), one of the first large-scale international surveys focusing on behavioral responses from the public starting from the early onset of COVID-19 pandemic in March 2020 until July 2021. This panel survey was conducted in 115 countries and regions with 64,427 participants in the baseline sample. We constructed country-level strength of family ties by extracting the first principal component of relevant WVS questions. Then we merged this country-level measure to individual-level PsyCorona survey by each respondent's country of residence. After combining PsyCorona survey with WVS-generated family ties, our study sample consists of 61,478 individuals from 93 countries. Table 1 presents the summary statistics of the study sample. We applied a random effects model to the data and estimated the association of country-level strength of family ties with individual non-pharmaceutical interventions compliance and vaccine adoption. We find that in countries with stronger family ties, people have greater propensity for complying with non-pharmaceutical interventions and receiving vaccines; additionally, young adults (age 18–34) would show more support towards mandatory vaccination. The associations between the strength of family ties and self-reported non-pharmaceutical interventions uptake and vaccine adoption were especially pronounced before the availability of COVID-19 vaccines, and they remain significant until the global vaccination coverage climbed beyond 25%.

**Table 1:** Descriptive statistics of study sample.

	Mean	SD	Min	Max	N
***Outcome variables***					
Hand-washing frequency	6.31	1.11	1	7	61,445
Avoid crowds	6.43	1.03	1	7	61,439
Self-quarantine	5.84	1.49	1	7	61,445
Vaccine for self	3.97	1.19	1	5	7859
Mandatory vaccination	5.28	1.84	1	7	61,446

***Independent variables***					
Family ties	−0.06	0.37	−0.99	0.83	61,478
Family ties (weighted)	−0.05	0.36	−0.98	0.83	61,478
Age categories					
18–24	22.61%				13,899
25–34	24.43%				15,018
35–44	19.15%				11,771
45–54	14.59%				8968
55–64	11.28%				6936
65–74	6.92%				4254
75–84	0.93%				569
85+	0.10%				63

Female	61.14%	0.49	0	1	61,478

High school education and above	75.67%	0.43	0	1	61,478

Unemployed	11.39%	0.32	0	1	61,478

Financially strained	1.98	1.21	0	4	61,478

Statistics for “Vaccine for self” was extracted from wave 4, when it was asked the first time. Statistics for all other variables were obtained from the baseline wave. Family ties (weighted) was calculated by assigning weights to the Family ties variable based on S017 in the WVS. The purpose of S017 is to account for small deviations in the sample in relation to key factors, which can be sex-age distribution, urban-rural distribution, or the distribution of respondent education.

Our analysis makes several contributions to the literature. First, our work contributes to the literature on the linkage between family ties and socioeconomic behaviors inspired by Alesina and Giuliano (2010). Their pioneering work and subsequent studies in economics, sociology and political sciences have shown that the strength of family ties is persistent overtime, and it is associated with a wide range of socioeconomic attitudes and outcomes, including generalized trust (Daniele & Geys, 2016; Ermisch & Gambetta, 2010), home and labor market production (Alesina et al., 2015; Alesina & Giuliano, 2010, 2014; Algan & Cahuc, 2005; Bertrand & Schoar, 2006; Daniele & Geys, 2016; Liu & Ren, 2021), political participation (Alesina & Giuliano, 2011, 2014) and quality of institution (Alesina & Giuliano, 2014; James Jr & Giwa-Daramola, 2023; Marè et al., 2020). We extend this line of literature by examining an additional outcome: willingness to uptake infection prevention measures in times of pandemic. As an extension to their construction of the family ties measure, our results also underscore the importance of customizing this measure to fit the research context of the COVID-19 pandemic.

Furthermore, we contribute to the emerging literature investigating the relationship between family dynamics and infection prevention during the COVID-19 pandemic. Utilizing within-family variations, scholars have shown that people's social distancing behaviors were affected by their family members' adherence (Aksoy, 2022), and that family members may relay misinformation which would aggravate elderly's misperception of the COVID-19 vaccines (Chia et al., 2023). Studies in this area have focused on one country or region. By utilizing a large panel dataset of 93 countries, we provide complementary cross-country results to illustrate the important role family ties as a cultural attribute plays in infection prevention.

Additionally, our work speaks to the literature on the cultural factors causing differences in public health compliance attitudes and behaviors in times of the pandemic. Deopa and Fortunato (2021) took advantage of Switzerland's multicultural geographic distribution to illustrate that cultural values contribute to difference in adherence to social distancing policies. In their study, regional cultural values were proxied by language used in the region. However, the question remains: which cultural values matter? Existing research provided some answers to this question, including trust in government (Bargain & Aminjonov, 2020), higher sense of civic duty (Barrios et al., 2021), and collectivism (Chen et al., 2021). Our study provides evidence that family ties may work as an additional mechanism motivating people to comply with public health measures. Moreover, most studies in the social distancing compliance literature during the COVID-19 pandemic focus on the short-term impacts. In contrast, PsyCorona survey's large sample size and broad geographical and temporal coverage allows us to investigate not only the association between country-level strength of family ties and individual willingness for infection prevention measures in the short-term, but also provides us with robust insight into the persistence of this relationship even after COVID-19 vaccines became available.

## 2 Materials and methods

### 2.1 Measure of the strength of family ties

We constructed country-level strength of family ties by using two questions from the latest wave of WVS launched in 2017 (Inglehart et al., 2022). Started in 1981, WVS is a collection of nationally representative surveys administered in almost 100 countries using a common questionnaire. Samples are representative of all residents aged 18 and older in private households in each country, and the sampling methods may be full probability or a combination of probability and stratified depending on the choice of each national survey team. For the main results, we used the latest WVS wave to capture contemporary strength of family ties in each country. Table S1 presents summary statistics of the WVS 2017 sample.

Respondents answered the two following questions by choosing a number from 1 to 4. The first question asked the respondents how important the family was in their life (1 is very important and 4 not important at all). And the second question asked the respondents whether one of her main goals in life had been to make her parents proud (1 is strongly agree and 4 is strongly disagree). We chose not to include some of the survey questions used by previous studies as they focused on parental duties and attitudes towards children.

For simplicity of results presentation, we first made adjustment so that higher values correspond to stronger family ties. We combined the answers to these two questions in the latest wave of WVS by extracting the first principal component. If a country did not participate in the 2017 wave, then we used the most recent wave available to calculate this country's family ties measure.

### 2.2 Measures of individual willingness to uptake infection prevention measures

Variables on individual self-reported uptake of nonpharmaceutical interventions and vaccine acceptance were extracted from five questions in PsyCorona survey (Agostini et al., 2022), a longitudinal multinational survey on the psychological and behavioral responses to the COVID-19 pandemic. The baseline surveys were conducted starting March 19th, 2020 in 115 countries and regions with 64,427 participants following the outbreak of COVID-19, and 22 follow-up surveys (weekly surveys through June 13th, 2020, and then monthly surveys) were conducted conditional on respondents’ willingness of continued participation through July 2021. Participants were recruited using convenience sampling, snowball sampling, and paid procedures. As such, PsyCorona does not provide nationally representative samples, a concern we address in the Validation and sensitivity analyses section.

### 2.3 Outcome variables and individual-level covariates

In total we considered five outcome variables: willingness to 1) increase handwashing frequency, 2) avoid crowded spaces, 3) self-quarantine, 4) get vaccinated and 5) support mandatory vaccination.

To assess uptake of a range of non-pharmaceutical interventions, starting from the baseline wave of PsyCorona survey, a set of three questions asked respondents whether they would agree with each of the following statements “to minimize the chances of getting coronavirus”: “I wash hands more often”, “I avoid crowded spaces”, and “I put myself in quarantine”. To gauge their support for mandatory vaccination, they were asked whether they would agree with the following statement: “I would sign a petition that supports mandatory vaccination once a vaccine has been developed for coronavirus”. To answer each question, respondents chose from a 7-point response scale (1: “strongly disagree”; 7: “strongly agree”).

In wave 4, respondents were asked for the first time, “How likely are you to get vaccinated against coronavirus once a vaccine becomes available?” To answer this question, respondents chose from a 5-point response scale (1: “Extremely unlikely”; 5: “Extremely likely”).

In addition, we obtained individual covariates from PsyCorona respondents at the time of the survey: age (measured in categories), gender, education level, employment status, whether they felt financially strained. However, we must note the limitation of this survey: it does not contain information on household members. Thus, though we believed the importance of household cohabiting pattern in health attitudes and behaviors, it was impossible for us to incorporate the following information into regression analysis: household size, number of children in the household, whether cohabiting with parents and/or in-laws.

Sample questionnaires of PsyCorona survey (baseline wave and wave 4) are included in the supplementary material.

### 2.4 Additional data sources for country-level measures

We obtained vaccination coverage and number of daily increases of confirmed cases from Our World in Data (Mathieu et al., 2020). Additionally, we obtained government stringency index from Oxford COVID-19 Government Response Tracker (OxCGRT), a composite daily measure based on 9 response indicators including school closures, workplace closures, and travel bans, whose final update was made in June 2023 (Hale et al., 2021). GDP per capita, out-of-pocket expenditure as percentage of current health expenditure in 2019 was obtained from Global Health Expenditure Database of World Health Organization (Global Health Expenditure Database). Institutional collectivism measure was obtained from the 2004 study of Global Leadership and Organization Behavior Effectiveness (GLOBE) project (House et al., 2004).

### 2.5 Estimation strategy

We examined the association between family ties and individual willingness to uptake preventative measures using the following specification with a random effects model:

$$Y_{ijt}=\alpha_{0}+\alpha_{1}{SFT}_{j}+X_{it}∙\Gamma +{\theta N}_{jt}+W_{j}∙\Pi +u_{ijt}$$

For each individual i in country j at survey wave t, $Y_{ijt}$ indicates respondent's willingness to uptake infection prevention measures. ${SFT}_{j}$ denotes the measure of country j's strength of family ties. $X_{it}$ is a vector of individual level covariates. $N_{jt}$ denotes a vector consists of 7-day average daily increase of confirmed cases per capita in country j one week prior to survey wave t and country j's government stringency index on the day of survey wave t. $W_{j}$ denotes two country-level measures in 2019 (or in the nearest year prior to 2019 with available data): natural log of GDP per capita, and out-of-pocket expenditure as percentage of health expenditure in 2019. Standard errors were clustered at the country level. For ease of interpretation, we present standardized regression coefficients.

For all estimation (Tables S2–S9), we also present standardized coefficients employing a version of family ties measure weighed by a WVS weight. For all analysis mentioned in the Validation and sensitivity analyses section, country-level vaccination coverage at time of the survey was controlled for.

### 2.6 Estimating time varying association

We estimated the time varying association of family ties with self-reported infection prevention measures uptake using the following specification:

$$Y_{ijt}=\beta_{0}+\beta_{1}\varphi *{SFT}_{j}+\beta_{2}{SFT}_{j}+X_{it}∙\Gamma +{\theta N}_{jt}+W_{j}∙\Pi +{VAC}_{jt}+u_{ijt}$$

Where φ is a vector of indicator variables denoting survey waves. The interaction of φ with SFT controls for the fact that the association of family ties and outcome variables could be weaker or stronger as time goes by, due to factors such as psychological fatigue towards the pandemic. ${VAC}_{jt}$ denotes country-level vaccination coverage at time of the survey wave. The sum of $\beta_{1}$ and $\beta_{2}$ measures the association at the time of a given survey wave, where the omitted category is the baseline wave (or the first wave when the outcome variable appeared in the survey).

## 3 Results

### 3.1 Strength of family ties measure by country (region)

**Fig. 1:**
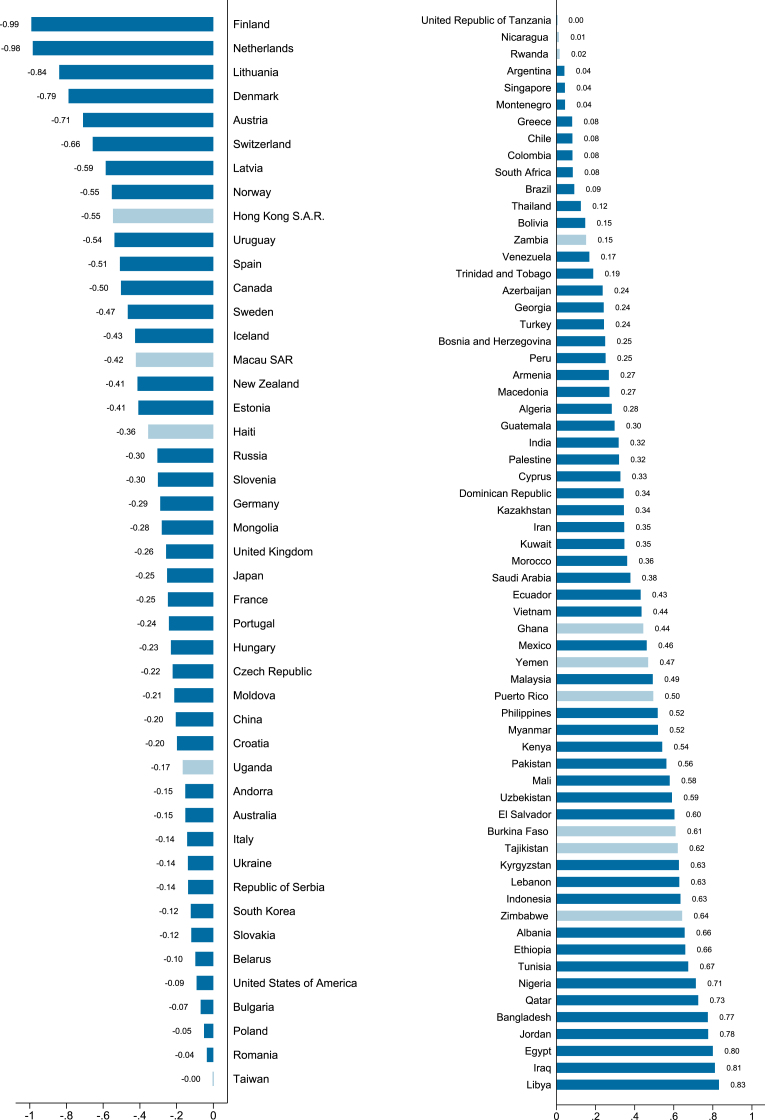
**Strength of family ties by country (region).** Higher values correspond to stronger family ties. This measure is constructed by using two questions from the latest available wave of data of the World Values Survey (WVS). The most recent wave of WVS was launched in 2017, when it was not available for a country, we substituted with data from the nearest available wave. The first question asked the respondents how important the family was in their life. And the second question asked the respondents whether one of her main goals in life had been to make her parents proud. We combined the answers to these two questions in the latest wave of WVS by extracting the first principal component. Light blue bars indicate countries and regions not included in subsequent analysis because no matching data is available in PsyCorona. (For interpretation of the references to color in this figure legend, the reader is referred to the Web version of this article.)

Fig. 1 displays the strength of family ties constructed by using questions from WVS launched in 2017. The rankings of this measure are consistent with public perceptions such that among the countries with the strongest family ties are African and Middle East countries, whereas northern European countries, Netherlands, Austria have the weakest family ties, and Asian countries mostly ranked in the middle. While the ranking is broadly similar to that in Alesina and Giuliano (2010), they are not identical. The differences may be attributed to two reasons – first, we constructed family ties measure differently by placing emphasis on how much people value their parents (instead of their attitudes towards the younger members of the family); second, we used a more recent wave of WVS.

### 3.2 Stronger family ties associated with more willingness to uptake non-pharmaceutical interventions and receive vaccines

**Fig. 2:**
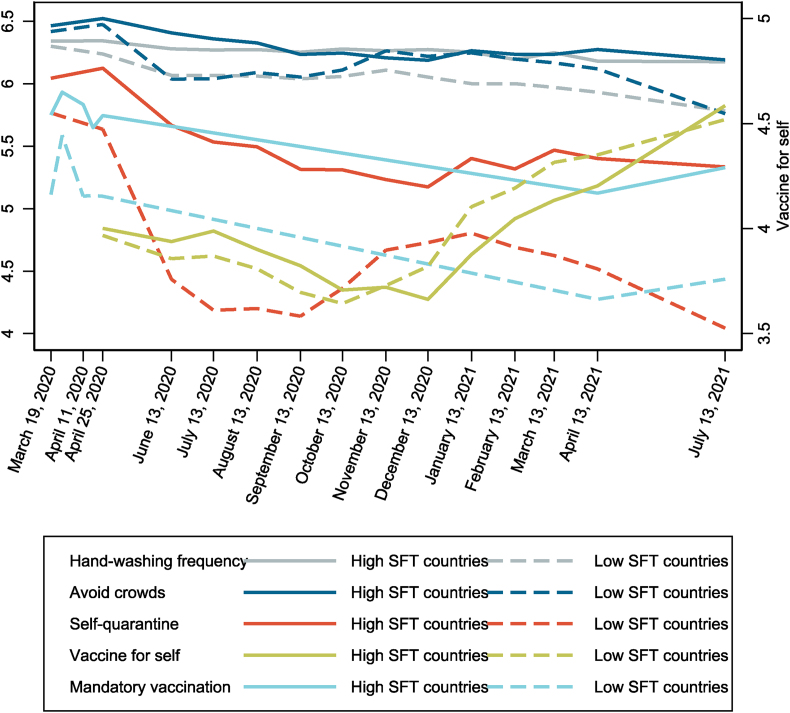
**Time trends of individual willingness towards infection prevention behaviors (raw data) from March 2020 to July 2021.** PsyCorona respondents indicate their willingness for each outcome on a 7-point response scale (and 5-point response scale for willingness to receive vaccines), where higher value corresponds to higher willingness (support). For each outcome, respondents are divided into two groups depending on whether the strength of family ties in their country exceeds (solid line) or below (dashed line) the median country-level strength of family ties. Data are for the entire study sample (n = 61,478).

Fig. 2 shows the evolution of respondents' self-reported uptake (raw data) of non-pharmaceutical interventions (increasing hand-washing frequency, avoiding crowded spaces, and self-quarantine) and vaccine acceptance (willingness to receive vaccine and support for mandatory vaccination). Countries are divided into two groups by the median country-level strength of family ties. Respondents from countries with stronger family ties showed greater inclination to uptake all three non-pharmaceutical interventions. Among the two mobility restricting measures, people showed less inclination to perform the more stringent measure of self-quarantine. Also, among countries with stronger family ties, a slight initial upward trend can be observed from March and April 2020 as the pandemic escalated. For all countries, there was generally less willingness to uptake non-pharmaceutical interventions as time passed by, which may be attributed to public's psychological fatigue towards the pandemic as well as the increase of vaccination coverage in 2021.

Amongst time trends for vaccine acceptance, the patterns between willingness to be vaccinated and support for mandatory vaccination are quite different. Similar to patterns of non-pharmaceutical interventions, strong family ties are associated with stronger support for mandatory vaccination throughout the time period examined, and an increase for support for mandatory vaccination can be observed between April to July 2021 in all countries. On the other hand, the increase in inclination to get vaccinated started from December 2020 when COVID-19 vaccines became available (Agency, 2020; Swissmedic, 2020, December 19, 2020). However, this increase was more pronounced among countries with weaker family ties.

**Fig. 3:**
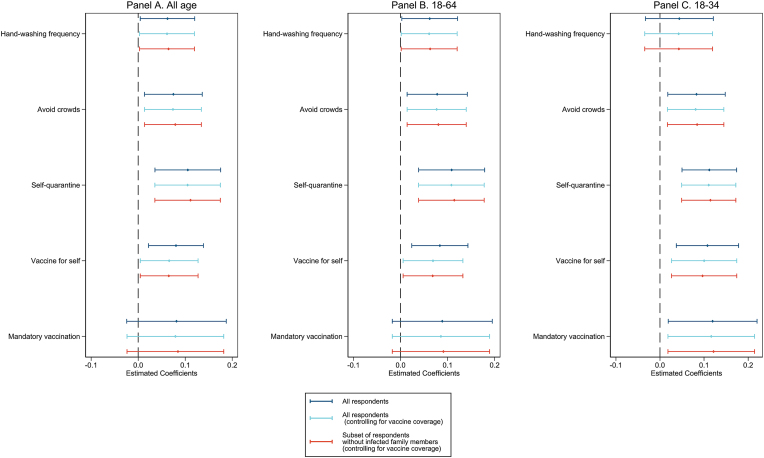
**Family ties and willingness to uptake infection prevention measures.** Each panel shows, for different age groups, the standardized regression coefficients of family ties with 95% confidence intervals for each of the five outcomes. All regressions controlled for individual covariates, country-level 7-day average daily increase of confirmed cases per capita one week prior to survey wave, country-level government stringency index on the day of the survey, natural log of GDP per capita in 2019, and out-of-pocket expenditure as percent of health expenditure in 2019. Standard errors were clustered at the country level. Numeric coefficients are also presented in Tables S2 and S3.

To examine associations between the strength of family ties and willingness to perform non-pharmaceutical interventions and vaccine acceptance, Fig. 3 reports standardized regression coefficients and 95% confidence intervals of the strength of family ties from random effects model (results also reported in Tables S2 and S3). Levels of infection risk, government policy stringency at the time of each survey wave were taken into consideration in addition to individual-level demographic information and country-level economic indicators. For each outcome, we show results for three different specifications: all respondents with and without adjusting for country-level vaccination coverage at the time of the survey, and subset of respondents without infected family members at the time of the survey. Fig. 3 Panel B and C also report coefficients for the working age subset (age 18–64) and subset of younger adults (age 18–34) respectively.

For all survey respondents (Fig. 3 A), willingness to comply with all three non-pharmaceutical interventions increase significantly with the country-level strength of family ties. And the coefficients increase in magnitude with the elevated stringency of the preventative measure. Specifically, one standard deviation (SD) increase in the strength of family ties is associated with 0.062 SD (s.e. = 0.030, P < 0.05) increase in willingness to increase hand-washing frequency, 0.075 SD (s.e. = 0.031, P < 0.05) increase in willingness to avoid crowd, and 0.106 SD (s.e. = 0.036, P < 0.01) increase in willingness to self-quarantine. For vaccine acceptance, the strength of family ties is highly significantly associated with willingness to get vaccinated (coeff. = 0.080, s.e. = 0.030, P < 0.01), whereas it does not have significant association with support for mandatory vaccination.

COVID-19 vaccines became available in December 2020 (Agency, 2020; Swissmedic, 2020, December 19, 2020). By July 13, 2021, 25.3% of the world population has received at least one dosage of COVID-19 vaccine (Mathieu et al., 2020). Vaccination coverage varies by country at any given date due to differences in vaccine availability and vaccine hesitancy. And country-level vaccination coverage may affect individual willingness to uptake infection prevention measures. For example, individuals may be less cautious with their own health behaviors when they perceive plenty of people around them have been vaccinated. Thus, we further control for country-level vaccination coverage in regressions and the coefficients of family ties are presented in light blue (see also Table S3). The significance of all family ties coefficients remains.

### 3.3 Stronger family ties associated with elevated willingness for infection prevention measures, especially when no family member was currently infected

To further illustrate the association between family ties and infection prevention compliance, we restricted regression subsamples to respondents whose family members (including themselves) were not infected with COVID-19 at the time of the survey. Coefficients of family ties are denoted by the color red in Fig. 3 (also see Table S2 Panel B and S3 Panel B). We performed the regression analysis with and without controlling for country-level vaccination coverage. For clarity, we only present coefficients controlling for country-level vaccination coverage graphically. Compared to their non-restricted counterparts, the family ties coefficients have similar statistical significance, but the magnitudes of most of the coefficients are slightly larger. In short, compared to the all-respondents sample, the strength of family ties has a stronger association with willingness to uptake infection prevention measures for this subset. This set of results is consistent with the scenario that individuals in countries with stronger family ties were extra careful in preventing a new infection when no one in their family was currently infected.

### 3.4 Higher support for mandatory vaccination amongst the young people in countries with stronger family ties

For the working age subset, the statistical significance of these results is identical to those of the main study sample, and the coefficients are slightly larger (Table S2, Panel A2). For the younger subset between age 18–34, the association between family ties and handwashing frequency is smaller and insignificant. However, it is worth noting that the association between family ties with the more restrictive forms of infection prevention measures (willingness to avoid crowd, self-quarantine, and receiving vaccination) are all larger and significant (P < 0.05). More importantly, young people in countries with stronger family ties show higher support for mandatory vaccination, and this association is statistically significant (coeff. = 0.119, s.e. = 0.051, P < 0.05). These results are consequential given the important role young people have played in within-family transmission during the pandemic. And mandatory vaccination is often associated with achieving herd immunity in policy discussions (Anderson et al., 2020).

### 3.5 Significant association between family ties and non-pharmaceutical intervention uptake before global vaccination coverage reached 25%

**Fig. 4:**
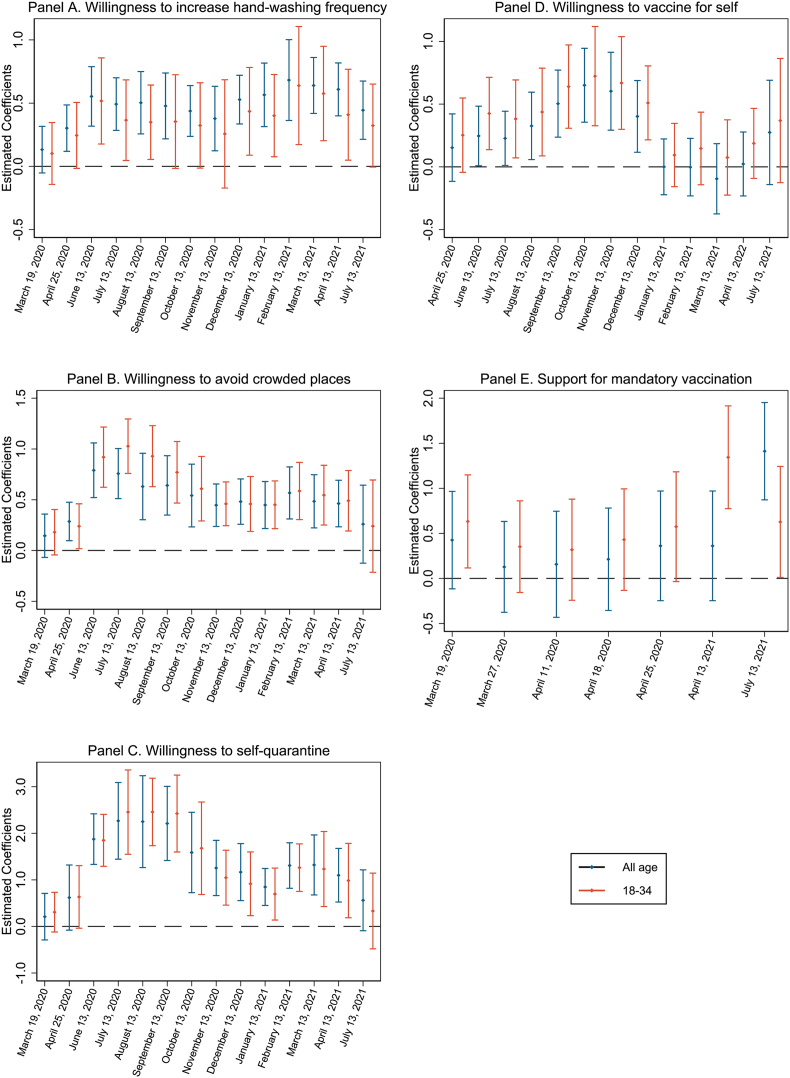
**Association between family ties and willingness to uptake infection prevention measures over time.** Each panel shows, for each of the five outcomes, the coefficients of family ties with 95% confidence intervals across survey waves. Results for the main study sample are drawn in black and those for the younger subset aged 18–34 is drawn in red. All regressions controlled for individual covariates, country-level 7-day average daily increase of confirmed cases per capita one week prior to survey wave, country-level government stringency index on the day of the survey, natural log of GDP per capita in 2019, out-of-pocket expenditure as percent of health expenditure in 2019, and country-level vaccination coverage at the time of the survey. Standard errors were clustered at the country level. (For interpretation of the references to color in this figure legend, the reader is referred to the Web version of this article.)

Additionally, to investigate the time trend of the association between family ties and willingness to uptake infection prevention measures, Fig. 4 reports coefficients of family ties across different survey waves adjusted for country-level vaccination coverage, level of infection risk, government policy stringency at the time of each survey wave, as well as individual-level demographic information and country-level economic indicators. Each panel reports the time varying association of family ties with a specific outcome; and within each panel, we report standardized coefficients on the all-age main study sample and age 18–34 subsample. Coefficients of the working age subsample are not presented graphically as we observed no substantial visual differences between their coefficients with those of the main study sample.

For handwashing frequency, consistent with the regression coefficients shown in Fig. 3, the coefficients of family ties are mostly significant for the main study sample, but they are persistently smaller and more likely to be insignificant for the 18–34 subset (Fig. 4 Panel A). The trends are very similar for the two mobility-restricting non-pharmaceutical interventions (Fig. 4 B, C): the magnitude of the association between family ties peaked in June and July 2020 respectively for these two outcomes. The coefficients are smaller, but remain significant after the availability of vaccines in December 2020 and before July 2021. Family ties coefficients for the younger subset peaked higher compared to the main study sample. Furthermore, there was stronger association between family ties and willingness to self-quarantine than to avoid crowds at this early stage of the pandemic. In summary, the associations between willingness to uptake each of the three non-pharmaceutical interventions with family ties are significant before the global vaccination coverage reached 25% in July 2021.

### 3.6 Family ties may motivate people to receive vaccines, except when vaccines are too new

The relationship between family ties and willingness to receive vaccines was mostly significant throughout 2020 (Fig. 4 Panel D). Interestingly, the tide has turned after the COVID-19 vaccines became a reality: the family ties coefficients became insignificant on willingness to receive vaccines between January and March 2021, and then became more positive in April and July 2021. One explanation is that caring for family members combined with safety concerns of new vaccines may motivate people to oppose receiving vaccines. As distrust towards new vaccines dissipated over time, the relationship between family ties and vaccine acceptance became positive once again. This finding is consistent with the non-trivial increases in country-level vaccination coverage (Fig. S1) as well as the significant positive correlation between the strength of family ties and the percentage increase of country-level vaccination coverage (p < 0.05) between April and July 2021 (Fig. S2).

As the survey question regarding support for mandatory vaccination is not asked in as many survey waves, the time trend in Fig. 4 E is not as clear. For the main study sample, we can observe generally positive albeit insignificant association between family ties in March and April 2020. Additionally, the coefficient of family ties became larger and significant in July 2021. This is consistent with a more positive change in attitudes towards vaccine adoption between April and July 2021 observed in Fig. 4 D. For both vaccine-related outcomes, the family ties coefficients are generally larger throughout all survey waves for age 18–34 subsample.

### 3.7 Validation and sensitivity analyses

This study faces several challenges. The primary challenges include that PsyCorona survey did not draw nationally representative samples from each country and the presence of sample attrition (See details in SI Text). These issues may undermine the external validity and internal validity of the study (Barry, 2005; Singh & Sagar, 2021). To ameliorate this concern, we reweighed the data by a combination of demographic variables, population scaled weight and non-response weight (See details of the weighting procedure in SI Text). We show regression results using weighted data in Table S4. And the coefficients we obtained after reweighting are even larger in magnitude and more significant compared to the unweighted regression results in Table S3. It should be noted that results in Table S4 are restricted to 31 countries given their balanced representation of all age groups in PsyCorona survey. But there is no statistical difference in the strength of family ties measure between these 31 countries and those not included in this analysis.

Another concern is potential endogeneity, given we cannot include country fixed effects in regression analysis as family ties are invariant at country level. To address this concern, we constructed two alternative measures of family ties. One alternative measure was constructed by only using WVS at least ten years prior to the start of the pandemic. Another alternative measure is the historical climate variability (See details in SI Text for measure construction). Both of these alternative measures are completely exogenous to contemporary individual attitudes and behaviors during the pandemic. We observe robust association between family ties and willingness to uptake infection prevention measures when using data from WVS 1995–2009 (Table S5). Previous research has established that regions with higher pre-industrial climatic variability displays a weaker family ties (Buggle & Durante, 2021). We also confirmed this finding: Table S6 Panel A shows the correlation coefficients between our measure of family ties and historical climate variability is −0.552, which is similar in magnitude with the correlation coefficients between historical climate variability and measure used by Alesina and Giuliano (2010). Since both family ties and historical climate variability are measured at the country level, we used historical climate variability as an alternative measure of family ties, instead of using it as an instrumental variable. Table S6 Panel B shows that higher historical climate variability (hence weaker family ties) is associated with lower willingness for non-pharmaceutical interventions and vaccine adoption. This set of results is not as robust, which is likely due to the fact that historical climate variability is still an imperfect replacement for the family ties measure despite their significant negative association. Also, it should be noted that results in Table S6 are restricted to 39 European countries for which we have historical climate data.

Additionally, one may be tempted to conclude that the association of willingness to uptake infection prevention measures and family ties shown in our study is no different than the effect of collectivism. Indeed, collectivism is intricately linked with family ties, given the construction of collectivism measure using survey data often containing questions on parents-children relationship (Brewer & Venaik, 2011; Guo et al., 2022). To distinguish the impact of family ties from that of a more broadly measured collectivism, we leverage the distinction between two collectivism measures, in-group collectivism and institutional collectivism in the GLOBE study (House et al., 2004). Brewer and Venaik recommended GLOBE's in-group collectivism to be relabeled as “family collectivism” for clarity, whereas these scholars considered “institutional collectivism” appropriately named given its reflection on leaders, groups and economic systems (Brewer & Venaik, 2011). Thus, we add GLOBE's institutional collectivism practices and values as additional country-level covariates to control for the part of collectivism measure that is not family-related, which would result in regression using 49 countries and regions. Fig. S4 shows that the association between family ties and all five outcomes remain robust for all three age-subsets. Additionally, both institutional collectivism social values and practices have significant coefficients (P < 0.01) on willingness to receive vaccines and support for mandatory vaccination for all three age-subsets. Additionally, the association between institutional collectivism social values and crowd-avoidance is significant for all three age-subsets (at least P < 0.05). Coefficients are also reported in Table S7.

Lastly, for potential endogeneity concerns with using concurrent country-level vaccination coverage, we used 7-day average of country-level vaccination coverage 30 days prior to each survey wave as an alternative control (Table S8). And we do not observe substantial differences between results in Table S3 and Table S8.

## 4 Discussion

This paper explored the relationship between cultural variation in the strength of family ties and individual willingness to uptake infection prevention measures in times of the global pandemic of COVID-19. With individual-level panel survey from 93 nations, we have shown that country-level strength of family ties, with an emphasis on people's perception of the importance of parents, has a significant positive association with individual willingness to uptake non-pharmaceutical interventions as well as vaccine acceptance. Controlling for country-level vaccination coverage, a one SD increase in the strength of family ties is associated with 0.061 SD increase in handwashing frequency (P < 0.05), 0.074 SD increase in crowd-avoidance (P < 0.05), 0.105 SD increase in self-quarantine (P < 0.01) and 0.066 SD increase in willingness to receive vaccines (P < 0.05). Additionally, compared to the all-age main analysis sample, the relationship between the strength of family ties and the two mobility-restricting non-pharmaceutical interventions and vaccine acceptance are stronger among the younger adults between age 18–34. Specifically, a one SD increase in the strength of family ties is associated with 0.081 SD increase in crowd-avoidance (P < 0.05), 0.111 SD increase in self-quarantine (P < 0.01) and 0.100 SD increase in willingness to receive vaccines (P < 0.01). Furthermore, among the younger adults, the strength of family ties is also significantly associated with support for mandatory vaccination (0.116 SD increase, P < 0.05). One potential reason is that compared to older people, a cultural norm of closer ties with parents may invoke even stronger reaction in infection prevention willingness in younger people, as their parents (and other elderly family members) are more likely to be alive and active at the time of the pandemic. Given the crucial role younger adults played in transmission through intergenerational contacts, this outcome holds significant importance.

One policy implication of this study is that messages on family values, issued along with scientific facts, may help to convince individuals to comply with infection prevention measures during future public health crises. The time trend results suggest that emphasizing family values can be an effective tool in promoting non-pharmaceutical interventions when they are needed the most - that is, before the mass adoption of vaccination. However, we have also observed suggestive evidence that family ties may work together with distrust in new vaccines to create vaccine hesitancy. It seems that in the early phase of a new vaccine release, more emphasis should be tilted towards publicity of vaccine safety, instead of family values. Our results also have practical implications for infection prevention of other communicable diseases and delivery of other newly developed vaccines.

The strength of this study lies in our combination of two large-scale surveys sampled from diverse geographic regions globally, which is crucial for examining the effect of country-level cultural attributes such as family ties. In particular, WVS provided us with consistent records over decades that makes measuring family ties possible. Spanning over the majority duration of the pandemic in which governments maintained elevated level of policy stringency (Fig. S3), monthly waves of PsyCorona panel survey shed light into the longitudinal perspective and helped us capture (changes in) individual attitudes and self-reported behaviors as the COVID-19 pandemic unfolded. Combining these two surveys enabled us to obtain robust results with a substantial number of participants from diverse cultural backgrounds; the multitudes of countries covered by these two surveys also boosts generalizability of the results.

Previous studies in the social distancing compliance literature during the COVID-19 pandemic focused on the short-term impacts. In contrast, our study follows individuals’ attitudes and self-reported behaviors after the COVID-19 vaccines became available, which enables us to explore the persistent association between family ties and infection prevention by parsing out the effect of vaccination coverage. Examining this relationship in longer-term is particularly important given the endurance of family ties in cultures and societies (Alesina & Giuliano, 2010). Moreover, our results derived from a panel dataset of 93 countries provide complementing evidence to studies using within-country and within-family variations (Aksoy, 2022). And our results are robust across several specifications, including when collectivism is taken into consideration.

In addition to extending the literature on the impact of cultural factors on public health compliance attitudes and behaviors during the COVID-19 pandemic, our study speaks to the literature on the association between family ties and socioeconomic behaviors. Much of the focus of this long line of literature has been on the juxtaposition of trust in family with the generalized trust in society. And the latter is associated with many positive collective outcomes. What sets our study apart is that we are able to show that positive outcomes, in this case health promoting behaviors, can arise from societies with high social trust (Borinca et al., 2021) as well as strong family ties.

Furthermore, to illustrate the difference between our measure of family ties and that from previous studies, we also constructed a family ties measure with the same three survey questions used by Alesina and Giuliano (2010) using WVS 1989–2010 waves (Table S9). In addition to rate how important family is, respondents were asked whether one must always love and respect parents and whether it is the parents' duty to do their best for their children, even at the expense of their own well-being. When using this family ties measure, the regression results are different. Notably, the significance of family ties coefficients on willingness to self-quarantine and willingness to get vaccinated has vanished altogether. To explore this point further, we also regressed all outcomes on each WVS question used in constructing family ties separately (Fig. S5). In contrast to children's attitudes towards parents, parental attitudes towards children (*parental duties*) does not have significant association with willingness to self-quarantine or willingness to get vaccinated.

## 5 Limitations

We should note limitations pertaining to our data: we could not obtain individual-level family ties measure from PsyCorona suvey given it did not ask questions on family values. As a result, this study can only investigate cross-country differences rather than within country variations. Also, PsyCorona survey did not document respondents’ immigration status, as such, we could not leverage this information to explore how people were affected by culture from their home countries. In addition, as respondents self-reported their willingness to perform non-pharmaceutical interventions, there may be deviation between self-reported and actual behaviors. On the other hand, given that people voluntarily took PsyCorona survey online, there seemed to be limited pressure on them to report inaccurately (Brenner & DeLamater, 2016).

## 6 Future research

Our research findings not only provide compelling evidence that family ties offer intrinsic motivation to uptake health measures to fend off a communicable disease, but also illustrate that it is meaningful to construct strength of family ties measure with specific emphasis. This may open up avenues for future research studying different aspects of family, including extended families and clans.

In addition, while there exists a large body of literature on using behavioral interventions to encourage vaccination, with few exceptions (Milkman et al., 2021; Reddinger et al., 2022), either family-related messages were not considered as a treatment or family tended to be mixed with community in treatment messages (Hong & Hashimoto, 2023; James et al., 2021). One possible reason is that past behavioral intervention studies were mostly conducted in developed economies where family ties were relatively weak (Reñosa et al., 2021), thus researchers did not perceive it as a priority. Future studies may wish to investigate the impact of family-values related nudges in a more diverse cultural context.

## 7 Conclusions

In summary, this study provides evidence that stronger country-level family ties were associated with more individual willingness to uptake non-pharmaceutical interventions and higher vaccine acceptance during the global health crisis of the COVID-19 pandemic. We also find that younger people exhibited higher support for mandatory vaccination in countries with stronger family ties. This association remained significant throughout the early phase of the pandemic until the global vaccination coverage reached 25%. Given the context that COVID-19 disproportionately affects older people, our empirical analysis finds it is meaningful to construct a strength of family ties measure with a focus on how children value their parents. This study contributes to a better understanding of the intricate link between family ties and health and suggests a potential mechanism behind the protective effects of social ties on health outcomes. Future public health policies may wish to incorporate cultural values as part of their infection prevention outreach.

## CRediT authorship contribution statement

**Ming Gu:** Writing – review & editing, Writing – original draft, Visualization, Validation, Project administration, Methodology, Investigation, Funding acquisition, Formal analysis, Data curation, Conceptualization.

## Declaration of competing interest

Author declares that she has no competing interests.

## Data Availability

PsyCorona baseline data is open access and subsequent waves are restricted and can be accessed by request through contact information provided at https://dataverse.nl/dataset.xhtml?persistentId=doi:10.34894/PX5IVZ. Analysis code and other publicly available data used in this study is available at https://doi.org/10.7924/r49z9c395.
