# Supplementary material for: The power of the family in times of pandemic: Cross-country evidence from 93 countries
Source: SSM Popul Health. 2024 Jul 4;27:101698. doi: 10.1016/j.ssmph.2024.101698 (PMC11320607; doi:10.1016/j.ssmph.2024.101698)
Supplement: Multimedia component 1 [file mmc1.pdf]

English: **Before starting the survey please keep in mind that you can change the language in the upper right corner. Please click on the arrow below to start the survey.**

Deutsch: **Bevor Sie die Umfrage starten, beachten Sie, dass Sie die Sprache oben rechts im Browserfenster ändern können. Bitte klicken Sie unten auf den Pfeil um den Fragebogen zu beginnen.**

Nederlands: **Houd er voor het starten van de vragenlijst rekening mee dat u de taal in de rechterbovenhoek kunt wijzigen Klik op de pijl hieronder om met de vragenlijst te beginnen.**

Italiano: **Prima di iniziare il sondaggio, tieni presente che puoi cambiare la lingua nell'angolo in alto a destra Clicca sulle freccia per iniziare il questionario.**

Français: **Avant de commençer l'enquête, veuillez noter que vous pouvez changer la langue dans le coin supérieur droit Veuillez cliquer sur la flèche ci-dessous pour commencer l'enquête.**

Español: **Antes de comenzar la encuesta tenga en cuenta que puede cambiar el idioma en la esquina superior derecha Por favor haga click en la flecha de abajo para empezar la encuesta**

عربى: **قبل بدء الاستبيان، الرجاء الأخذ بالعلم أنّه بإمكانك تغيير اللّغة في الزاوية اليمنى العليا. من فضلك اضغط على السهم الموجود أدناه لبدء الاستبيان.**

فارسی: **به خاطر داشته باشید که شما میتوانید زبانِ پرسشنامه را با کلیک کردن بر روی دکمهٔ بالای سمت راست صفحه عوض کنید. برای شروع کردن، لطفا روی فلش پأیین صفحه کلیک کنید.**

Tiếng Việt: **Trước khi bắt đầu trả lời các câu hỏi, xin hãy nhớ rằng anh/chị có thể lựa chọn ngôn ngữ ở ô phía trên góc phải**

Bahasa Indonesia: **Sebelum survei ini dimulai, Anda bisa mengubah Bahasa yang digunakan dalam survei ini di pojok kanan atas. Silahkan klik tanda panah lanjut untuk memulai survei**

Bahasa Malaysia: Sebelum memulakan soal selidik, diingatkan bahawa anda boleh menukar bahasa di bahagian sudut kanan atas.

Türkçe: **Çalışmaya başlamadan önce anket dilini sağ üst köşeden Türkçe olarak seçebilirsiniz. Ankete başlamak için lütfen aşağıdaki ok işaretine tıklayınız.**

Polski: Przed rozpoczęciem ankiety pamiętaj, że możesz zmienić język w prawym górnym rogu. Kliknij strzałkę poniżej, aby rozpocząć ankietę.

Українська: **Перш ніж розпочати опитування, пам’ятайте, що Ви можете змінити мову у верхньому правому куті. Натисніть знизу на стрілку, щоб розпочати опитування.**

Русский: **Перед началом опроса имейте в виду, что язык можно изменить в правом верхнем углу. Нажмите на стрелку внизу, чтобы начать опрос.**

Ελληνικά: **Πριν ξεκινήσετε, λάβετε υπόψη ότι μπορείτε να αλλάξετε τη γλώσσα στην επάνω δεξιά γωνία. Παρακαλούμε πατήστε στο βέλος που θα βρείτε παρακάτω για να ξεκινήσετε την έρευνα.**

ไทย: ก่อนเริ่มการสำรวข กรุณาระลึกไว้ว่าคุณสามารถเปลี่ยนภาษาได้ที่บริเวณมุมขวาบน กรุณาคลิ้กลูกศรด้านล่างเพื่อเริ่มการสำรวจ

日本人: この調査を始める前に、ページの右上から言語を変更することができます。 下の矢印をクリックして調査を開始してください。

한국어: **설문 조사를 시작하기 전에 오른쪽 상단에서 언어를 변경할 수 있습니다. 설문을 시작하시려면 아래 화살표를 눌러주시기 바랍니다.**

中文: 開始進行調查之前，請記住您可以在右上角更改語言 請點擊下面的箭咀開始問卷調查。

中文: 在开始进行调查之前，您可以在右上角更改语言选项 请点击下面的箭头开始问卷调查。

Српски: **Пре почетка истраживања имајте на уму да можете да промените језик у горњем десном углу. Кликните на стрелицу испод да бисте почели са истраживањем.**

Hrvatski: **Prije nego pristupite istraživanju imajte na umu da možete promijeniti jezik u gornjem desnom kutu. Da biste započeli s istraživanjem, molimo Vas da pritisnete strelicu na dnu stranice.**

Română: **Înainte de a începe să participați la studiu, vă rugăm să țineți minte faptul că puteți oricând schimba limba din colțul din dreapta sus a ecranului. Vă rugăm să dați click pe săgeata de mai jos pentru a începe studiul.**

Português(esa): **Antes de iniciar a pesquisa, lembre-se de que você pode alterar o idioma no canto superior direito. Por favor carregue na seta em baixo para comecar a pesquisa.**

हिन्दी: सर्वे शुरू करने से पहले कृपया ध्यान रखें कि आप ऊपरी दाएं कोने में भाषा बदल सकते हैं। सर्वे शुरू करने के लिए कृपया नीचे दिए गए तीर पर क्लिक करें।

Magyar: Mielőtt elkezdi kitölteni a kérdőívet ne felejtse el a nyelvet magyarra állítani a jobb felső sarokban! Kérjük, kattintson a nyílra a kérdőív megkezdéséhez.

Shqip: Para se të filloni sondazhin, ju lutem keni parasysh se mund ta ndrroni gjuhën në këndin e sipërm në të djathtë. Ju lutem klikoni në shigjetën më poshtë për të filluar anketimin.

Informed consent Psycorona study

Principal Investigators: Dr. N. P. Leander, University of Groningen & Dr. J. J. Bélanger, New York University Abu Dhabi Ethics approval: ecp@rug.nl (study code: PSY-1920-S-0390); irbnyuad@nyu.edu (study code: HRPP-2020-42)

Purpose of the research
Thank you for volunteering. This survey will ask about your thoughts, feelings, and motivations regarding recent developments in society, the coronavirus, and your current economic situation. This survey typically takes 20 minutes. If you decide to complete this survey, you can decide to be recontacted during the coronavirus outbreak for more surveys as part of the same study. This will help us understand how human beings deal with the current situation.

You are under no obligation to participate in this survey. If you agree to participate, you are free to stop your participation at any time by closing the browser window. Your data will then be removed from the data set.

Types of data collected
This is a list of sensitive questions you will encounter in this survey. Note that you can always skip any question you do not feel comfortable answering. If you are interested in understanding how we protect your privacy, personal data, and rights have a look here.
Personal data (optionally provided by you)
Directly identifiable personal data:
- Email address, location data (i.e., postal code)
Indirectly identifiable personal data:
- Gender, age range, education level, employment status, country of residence
Sensitive personal data (a.k.a. special categories of personal data)
- Political beliefs, religious beliefs, health data

How will data be collected and handled
We have specified a full list of steps on how we handle your data and protect your privacy. Especially your directly identifiable data is protected through a rigorous mechanism. If you want to see the specific steps of how we handle your data, have a look here.

Please note that developmental research studies are never completely anonymous. We will need to collect a means of contacting interested participants for follow-up surveys and will generate a random participant identification number in order to connect data over multiple measurements.
However, we have taken the following steps to protect your privacy:
(1) If you choose to be invited to follow-up surveys we will ask you to provide an email address. Your email address and location data (postal code) are considered “directly identifiable personal data”. We will delete your email address and postal code within three months of the project completion or by January 1st, 2021 (if the study extends beyond ethical approval (i.e., January

1st, 2021), we will extend it at the consent of the ethics board; this coming fall 2020, we will do a data protection impact assessment to determine if the information is still up to date and we will take more measures if needed; we will make all that information available on our website). Your email address will also only be used for recontact and data merging. We will remove your email from the data sets after the data provided in different surveys have been linked. The data set is then effectively pseudonymized.
(2) We also ask you to optionally provide “sensitive personal data” (i.e., your religious and political beliefs, and health information; also see GDPR) as well as some “indirectly identifiable personal data” (i.e., your gender, mother language, education level, and country of residence). We collect these data in order to learn more about individual and group differences in dealing with the corona virus around the world. However, to minimize access to your sensitive and personal data the joined controller group has put a proposal system in place where access to the data is only granted upon vetted requests and only to variables absolutely necessary to the research question of collaborating researchers (see here for the full process).
(3) The data from this study will be stored in a secure location in the Department of Psychology at the Universities pursuant to data management policies of the universities and regulations of the federal government and the American Psychological Association and the Netherlands Ethics Code (Nethics code).
(4) Only members of the research team will have access to the survey data.
(5) You have the right to obtain a copy of your data, correct it or ask to delete it until January 1st, 2021.
(6) If you would like to know more about how exactly your data will be processed and shared with collaborators see our data handling documentation and our Frequently Asked Questions section on our website. The data will be used solely for scientific and public information purposes, but will not be used for commercial purposes.

Rights of participants
If you have any questions about your rights, do not hesitate to contact privacy@rug.nl or the data protection officer at a.r.deenen@rug.nl (A.R. Deenen).

Information about storage and re-use of data
We have developed a full data management procedure about where your data is stored and how long it is stored. There you can also have a look at how we share your data with researchers from other universities.

I have read the above information. I agree to participate in this study and to the processing of my personal data as described above. I also agree to my data being transferred outside of the European Economic Area. I understand that my participation is entirely voluntary and that I may withdraw at any time by closing the browser window. I know that I can ask to have my data accessed, changed, or erased.

Do you agree to participate in this study (and confirm that you are older than 18)?

○ Yes, I agree to participate.

We will start by asking some general questions about yourself.

In which country do you currently live in? Please answer the question keeping in mind the country you spend most of the year in. Please write this country into the following field.

____

How did you feel over the last week?

	Very slightly or not at all	A little	Moderately	Quite a bit	Extremely
Anxious	○	○	○	○	○
Bored	○	○	○	○	○
Calm	○	○	○	○	○
Content	○	○	○	○	○
Depressed	○	○	○	○	○
Energetic	○	○	○	○	○
Excited	○	○	○	○	○
Nervous	○	○	○	○	○
Exhausted	○	○	○	○	○
Inspired	○	○	○	○	○
Relaxed	○	○	○	○	○

How likely is it that the following will happen to you in the next few months?

	Exceptionally unlikely	Very Unlikely	Unlikely	About equally likely	Likely	Very likely	All but certain	Already happened
You will get infected with coronavirus.	○	○	○	○	○	○	○	○
Your personal situation will get worse due to economic consequences of coronavirus.	○	○	○	○	○	○	○	○

Agree or disagree:

	Strongly disagree	Disagree	Neither agree nor disagree	Agree	Strongly agree
I fear that things will go wrong in society.	○	○	○	○	○
I feel concerned when I think about the future of society.	○	○	○	○	○
I am satisfied with society.	○	○	○	○	○

Agree or disagree:

	Strongly disagree	Disagree	Neither agree nor disagree	Agree	Strongly agree	(Not applicable)
Chances are, I will soon lose my job.	○	○	○	○	○	○
I am sure I can keep my job.	○	○	○	○	○	○
I feel insecure about the future of my job.	○	○	○	○	○	○
I already lost my job.	○	○	○	○	○	○

Agree or disagree:

	Strongly disagree	Disagree	Neither agree nor disagree	Agree	Strongly agree
I am financially strained.	◯	◯	◯	◯	◯
I often think about my current financial situation.	◯	◯	◯	◯	◯
Due to my financial situation, I have difficulties paying for my expenses.	◯	◯	◯	◯	◯

Agree or disagree:

	Strongly disagree	Disagree	Neither agree nor disagree	Agree	Strongly agree
Not a lot is done for people like me in this country (**[country Choice]**).	◯	◯	◯	◯	◯
If I compare people like me against other people in this country (**[country Choice]**), my group is worse off.	◯	◯	◯	◯	◯
Recent events in society have increased my struggles in daily life.	◯	◯	◯	◯	◯

In the past 7 days, how much social contact have you had with <u>people who live outside your household</u>?

	In the past 7 days, how many days did you have **in-person** (face-to-face) contact with ...								In the past 7 days, how many days did you have **online** (video or voice) contact with ...							
	0	1	2	3	4	5	6	7	0	1	2	3	4	5	6	7
...friends or relatives	○	○	○	○	○	○	○	○	○	○	○	○	○	○	○	○
...other people in general	○	○	○	○	○	○	○	○	○	○	○	○	○	○	○	○
...immigrants	○	○	○	○	○	○	○	○	○	○	○	○	○	○	○	○

The following questions are about your personal relationships.

In general, how satisfied are you with your personal relationships?

- ○ 1 Extremely unsatisfied
- ○ 2
- ○ 3
- ○ 4
- ○ 5
- ○ 6
- ○ 7
- ○ 8
- ○ 9
- ○ 10 Extremely satisfied

Do you have anyone with whom you can discuss very personal matters?

- ○ Yes
- ○ No
- ○ I don't know

The following questions are about how you felt during the past week.

During the past week, did you...

	Never	Rarely	Sometimes	Often	All the time
...feel **lonely**?	◯	◯	◯	◯	◯
...feel **isolated** from others?	◯	◯	◯	◯	◯
...feel **left out**?	◯	◯	◯	◯	◯

To what extent do you think that the country you currently live in ([country Choice]) should have the following characteristics right now?

	1	2	3	4	5	6	7	8	9	
1: Have flexible social norms	◯	◯	◯	◯	◯	◯	◯	◯	◯	9: Have rigit social norms
1: Be loose	◯	◯	◯	◯	◯	◯	◯	◯	◯	9: Be tight
1: Treat people who don’t conform to norms kindly	◯	◯	◯	◯	◯	◯	◯	◯	◯	Treat people who don’t conform to norms harshly

In general, how happy would you say you are?

- ○ 1 Extremely unhappy
- ○ 2
- ○ 3
- ○ 4
- ○ 5
- ○ 6
- ○ 7
- ○ 8
- ○ 9
- ○ 10 Extremely happy

In general, how satisfied are you with your life?

- ○ Very dissatisfied
- ○ Dissatisfied
- ○ Slightly dissatisfied
- ○ Slightly satisfied
- ○ Satisfied
- ○ Very satisfied

Agree or disagree:

"**My life has a clear sense of purpose."**

- ○ Strongly disagree
- ○ Disagree
- ○ Somewhat disagree
- ○ Neither agree nor disagree
- ○ Somewhat agree
- ○ Agree
- ○ Strongly agree

When dealing with stressful situations, what do you usually do?

	(Almost) never	Rarely	Sometimes	Often	(Almost) always
I try to come up with a strategy about what to do.	○	○	○	○	○
I make a plan of action.	○	○	○	○	○
I think hard about what steps to take.	○	○	○	○	○

When dealing with stressful situations, what do you usually do?

	(Almost) never	Rarely	Sometimes	Often	(Almost) always
I distract myself to avoid thinking about the subject.	○	○	○	○	○
I do things to distract myself from my thoughts and feelings.	○	○	○	○	○
I force myself to think about something else.	○	○	○	○	○

The next questions are about your experiences with the coronavirus outbreak.

How knowledgeable are you about the recent outbreak of Covid-19, commonly referred to as the Coronavirus, in this country ([country Choice])?

- ○ Not at all knowledgeable
- ○ Slightly knowledgeable
- ○ Moderately knowledgeable
- ○ Very knowledgeable
- ○ Extremely knowledgeable

Agree or disagree:

	Strongly disagree	Disagree	Somewhat disagree	Neither agree nor disagree	Somewhat agree	Agree	Strongly agree
I have high hopes that the situation regarding coronavirus will improve.	○	○	○	○	○	○	○
I think that this country (**[country Choice]**) is able to fight the Coronavirus.	○	○	○	○	○	○	○

Agree or disagree:

"I am willing to..."

	Strongly disagree	Disagree	Somewhat disagree	Neither agree nor disagree	Somewhat agree	Agree	Strongly agree
...help others who suffer from coronavirus.	○	○	○	○	○	○	○
...make donations to help others that suffer from coronavirus.	○	○	○	○	○	○	○
...protect vulnerable groups from coronavirus even at my own expense.	○	○	○	○	○	○	○
...make personal sacrifices to prevent the spread of coronavirus.	○	○	○	○	○	○	○

Agree or disagree:

"To minimize my chances of getting coronavirus, I..."

	Strongly disagree	Disagree	Somewhat disagree	Neither agree nor disagree	Somewhat agree	Agree	Strongly agree
...wash my hands more often.	○	○	○	○	○	○	○
...avoid crowded spaces.	○	○	○	○	○	○	○
...put myself in quarantine.	○	○	○	○	○	○	○

Agree or disagree:

"I would sign a petition that supports..."

	Strongly disagree	Disagree	Somewhat disagree	Neither agree nor disagree	Somewhat agree	Agree	Strongly agree
...mandatory vaccination once a vaccine has been developed for coronavirus.	○	○	○	○	○	○	○
...mandatory quarantine for those that have coronavirus and those that have been exposed to the virus.	○	○	○	○	○	○	○
...reporting people who are suspected to have coronavirus.	○	○	○	○	○	○	○

Do you personally know anyone who <u>currently</u> has coronavirus?
(click all that apply)

- ☐ Yes, myself
- ☐ Yes, a member of my family
- ☐ Yes, a close friend
- ☐ Yes, someone I know
- ☐ Yes, someone else
- ☐ No, I do not know anyone

Agree or disagree:

"Right now, people in my area..."

	Strongly disagree	Disagree	Somewhat disagree	Neither agree nor disagree	Somewhat agree	Agree	Strongly agree
...**should** self-isolate and engage in social distancing.	○	○	○	○	○	○	○
...**do** self-isolate and engage in social distancing.	○	○	○	○	○	○	○

To what extent is your community….

	1: Not at all	2	3	4	5	6: Very much
...developing strict rules in response to the Coronavirus?	○	○	○	○	○	○
...punishing people who deviate from the rules that have been put in place in response to the Coronavirus?	○	○	○	○	○	○
...well organized in responding to the Coronavirus?	○	○	○	○	○	○

To what extent….

	1: Messages are completely unclear/ ambiguous	2	3	4	5	6: Messages are very clear/ unambiguous
...are you getting clear, unambiguous messages about what to do about the Coronavirus?	○	○	○	○	○	○

How personally disturbing would you find the following possible consequences of the coronavirus?

	1: Not disturbing at all	2	3	4	5: Extremely disturbing
Me contracting the virus	○	○	○	○	○
Me suffering negative economic consequences	○	○	○	○	○
Cancellation of my plans	○	○	○	○	○
Changing my life's routines	○	○	○	○	○

The next questions are about your experiences with the economic and financial consequences of coronavirus.

How knowledgeable are you about the potential economic and financial consequences of coronavirus in this country (**[country Choice]**)?

○ Not at all knowledgeable

○ Slightly knowledgeable

○ Moderately knowledgeable

○ Very knowledgeable

○ Extremely knowledgeable

Agree or disagree:

	Strongly disagree	Disagree	Somewhat disagree	Neither agree nor disagree	Somewh at agree	Agree	Strongly agree
I have high hopes that the situation regarding the economic and financial consequences of coronavirus will improve.	○	○	○	○	○	○	○
I think that this country (**[country Choice]**) is able to fight the economic and financial consequences of coronavirus.	○	○	○	○	○	○	○

Indicate your agreement or disagreement with the following statements.

To help with the economic and financial consequences of coronavirus, I am willing to...

	Strongly disagree	Disagree	Somewhat disagree	Neither agree nor disagree	Somewhat agree	Agree	Strongly agree
...help others who suffer from such consequences.	○	○	○	○	○	○	○
...make donations to help others that suffer from such consequences.	○	○	○	○	○	○	○
...protect vulnerable groups from such consequences, even at my own expense.	○	○	○	○	○	○	○
...make personal sacrifices.	○	○	○	○	○	○	○

Indicate your agreement or disagreement with the following statements.

If it would alleviate the economic and financial consequences of coronavirus, I would sign a petition that supports...

	Strongly disagree	Disagree	Somewhat disagree	Neither agree nor disagree	Somewhat agree	Agree	Strongly agree
...higher taxes.	○	○	○	○	○	○	○
...giving the government more authority over people.	○	○	○	○	○	○	○
...increased government spending.	○	○	○	○	○	○	○

The next questions are about your overall experiences.

In the past week, how often did you leave your home?

- ○ I did not leave my home.
- ○ Once or twice.
- ○ Three times.
- ○ Four times or more.

In the previous question, you have indicated that in the past week, you left the home at least once.

What were the reasons for leaving your home?
(Select all that apply)

- ☐ I had to go to work.
- ☐ I had errands to run.
- ☐ For leisure purposes with others (e.g., meeting up with friends, seeing family, going to the cinema, etc.)
- ☐ For leisure purposes alone (e.g., running, going for a walk, etc.)
- ☐ Other, please specify: ____

Indicate your agreement or disagreement with the following statements.

	Strongly disagree	Disagree	Somewhat disagree	Neither agree nor disagree	Somewhat agree	Agree	Strongly agree
I wish time would go by faster.	○	○	○	○	○	○	○
Time is moving very slowly.	○	○	○	○	○	○	○
I feel in control of my time.	○	○	○	○	○	○	○

Agree or disagree:

	Strongly disagree	Disagree	Somewhat disagree	Neither agree nor disagree	Somewhat agree	Agree	Strongly agree
I replay memories of the past in my mind.	○	○	○	○	○	○	○
I focus on what is currently happening in my life.	○	○	○	○	○	○	○
I think about what my future has in store.	○	○	○	○	○	○	○

When you think about your contacts with immigrants last week, do these interactions prove to be helpful or hindering?

○ 0 hindering

○ 1

○ 2

○ 3

○ 4

○ 5

○ 6

○ 7

○ 8

○ 9

○ 10 helpful

Thinking about your contacts with immigrants, how positive or negative did you experience them to be?

- ○ 0 Extremely negative
- ○ 1
- ○ 2
- ○ 3
- ○ 4
- ○ 5
- ○ 6
- ○ 7
- ○ 8
- ○ 9
- ○ 10 Extremely positive

Migrants who come to live here, generally...

	1	2	3	4	5	6	7	8	9	10	
take jobs away	○	○	○	○	○	○	○	○	○	○	create new jobs
undermine the cultural life	○	○	○	○	○	○	○	○	○	○	enrich the cultural life
make crime problems worse	○	○	○	○	○	○	○	○	○	○	make crime problems better
harm this country's (**[country Choice]**) culture	○	○	○	○	○	○	○	○	○	○	benefit this country's (**[country Choice]**) culture
bring diseases	○	○	○	○	○	○	○	○	○	○	help prevent diseases

Below is a feeling thermometer.
Ratings between 50 degrees and 100 degrees mean that you feel favorable and warm toward the group. Ratings between 0 degrees and 50 degrees mean that you don't feel favorable toward the group and that you don't care too much for that group. You would rate the group at the 50 degree mark if you don't feel particularly warm or cold toward the group.

How would you rate: Migrants who come to live here

- ○ 100˚ Very warm or favorable feeling
- ○ 85˚ Quite warm or favorable feeling
- ○ 70˚ Fairly warm or favorable feeling
- ○ 60˚ A bit more warm or favorable feeling than cold feeling
- ○ 50˚ No feeling at all
- ○ 40˚ A bit more cold or unfavorable feeling than warm feeling
- ○ 30˚ Fairly cold or unfavorable feeling
- ○ 15˚ Quite cold or unfavorable feeling
- ○ 0˚ Very cold or unfavorable feeling

Please answer the following questions.

You are presented with two policy options to combat the spread of coronavirus. Your public health agency will enact one of the policies. Policy A has a 95% chance to prevent 1000 new infections. Policy B has a 90% chance to prevent 5000 new infections. Which policy should your health agency enact to prevent the most infections?

- ○ Policy A
- ○ Policy B

Does the conclusion follow logically from the premises?   Premises: - All infected people cough and have a fever - Your neighbor coughs and has a fever   Conclusion: Your neighbor is infected

- ○ Conclusion follows logically from the premises
- ○ Conclusion does not follow logically from the premises

Imagine that every time you leave your home you have a 50/50 chance of getting infected with coronavirus. You have left your home 5 times recently and nothing has happened. The next time you leave your home, do you think that:

- ○ It is more likely that you will get infected
- ○ It is more likely that you will <u>not</u> get infected
- ○ getting infected and not getting infected are equally likely

Choose the picture which best represents the closeness between the people of the country you currently live in ([country Choice]) and yourself.

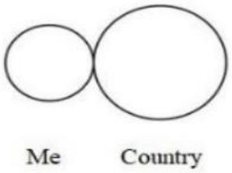

○

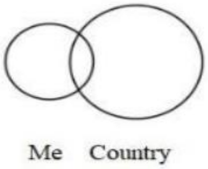

○

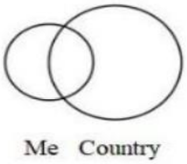

○

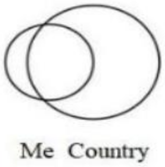

○

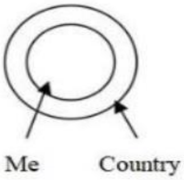

○

Indicate your agreement or disagreement with the following statements.

I see myself as someone who...

	Strongly disagree	Disagree	Somewhat disagree	Neither agree nor disagree	Somewhat agree	Agree	Strongly agree
...is very concerned.	○	○	○	○	○	○	○
...easily gets nervous.	○	○	○	○	○	○	○
...is relaxed, can easily deal with stress.	○	○	○	○	○	○	○

The following questionnaire deals with thoughts and feelings that one may experience in certain situations.
For each of the feelings and thoughts described below, please indicate how much they apply to you right now from 0 (not at all) to 10 (very much).
Feel free to answer based on what first came to mind. There are no right or wrong answers.

	Not at all	1	2	3	4	5	6	7	8	9	Very much
I need to be on my guard against others	○	○	○	○	○	○	○	○	○	○	○
People are trying to make me upset	○	○	○	○	○	○	○	○	○	○	○
Strangers and friends look at me critically	○	○	○	○	○	○	○	○	○	○	○

I think that......

	Certainly not 0%	10%	20%	30%	40%	Undecided 50%	60%	70%	80%	90%	Certainly 100%
… many very important things happen in the world, which the public is never informed about.	○	○	○	○	○	○	○	○	○	○	○
… politicians usually do not tell us the true motives for their decisions.	○	○	○	○	○	○	○	○	○	○	○
… government agencies closely monitor all citizens.	○	○	○	○	○	○	○	○	○	○	○

To what extent do you want more money than time?

○ 1: Much more time than money

○ 2

○ 3

○ 4: Equal amounts of both

○ 5

○ 6

○ 7: Much more money than time

To what extent do you want more time than money?

- ○ 1: Much more money than time
- ○ 2
- ○ 3
- ○ 4: Equal amounts of both
- ○ 5
- ○ 6
- ○ 7: Much more time than money

Rank order the following values in terms of the value you accord them
(1 = I value this the most; 6 = I value this the least)

(Click-and-drag the items to rank order them

Achievement
Creativity
Innovation
Sacrifice
Cooperation
Altruism

Rank order the following professions in terms of your appreciation of them
(1 = I appreciate this profession the most; 6 = I appreciate this profession the least)

(Click-and-drag the items to rank order them)

Scientists
Politicians
Athletes
Entertainers
Health professionals
Businesspeople

Rank order the following in terms of their importance in life
(1 = Very important in my life; 6 = Not very important in my life)

(Click-and-drag the items to rank order them)

Beauty
Achievement
Victory
Friendship
Love
Empathy

Next are some personal questions. Please know that you can skip any question you feel uncomfortable answering.

Are you religious?

○ Yes

○ No

What is your religion?

____

Do you believe in a God or Gods?

○ Yes

○ No

○ Unsure

Agree or disagree:

	Strongly disagree	Disagree	Somewhat disagree	Neither agree nor disagree	Somewhat agree	Agree	Strongly agree
God is sovereign and in complete control of our lives.	○	○	○	○	○	○	○
God can be influenced by our actions and our prayers.	○	○	○	○	○	○	○

For each of the following traits, please indicate how characteristic you think each trait is of **the god or gods you believe in**. If you believe in more than one god or deity, please respond based on one that is particularly important to you.

	1 (Not at all characteristic)	2	3	4	5	6 (Entirely Characteristic)
Punishing	○	○	○	○	○	○
Forgiving	○	○	○	○	○	○

In general, how much do you trust each of the following to take the right measures to deal with the coronavirus pandemic?

	Not at all	A little	A moderate amount	A lot	A great deal
The government of your country	○	○	○	○	○
Your community	○	○	○	○	○

Finally, we would like to ask you about some demographic information. Please know that you can skip any question you feel uncomfortable answering.

Are you a citizen of this country ([country Choice])?

- ○ Yes
- ○ No

Which country are you a citizen of? Please enter the name.

____

Have you been a citizen of this country ([country Choice]) since birth?

- ○ No
- ○ Yes

Do you consider yourself to be an immigrant?

- ○ No
- ○ Yes

Which of the following categories best describes your employment status <u>during the last week</u> (multiple may apply)?

- ☐ Employed, working 1-24 hours per week
- ☐ Employed, working 24-39 hours per week
- ☐ Employed, working 40 or more hours per week
- ☐ Not employed, looking for work
- ☐ Not employed, **not** looking for work
- ☐ Homemaker
- ☐ Retired
- ☐ Disabled, not able to work
- ☐ Student
- ☐ Volunteering

In a typical work day, what percentage of your <u>paid</u> work time did you complete from home?

	0 10 20 30 40 50 60 70 80 90 100
Percentage of work completed from home	

In a typical work day, how often do you interact with others at work as part of your job?

- ○ Never
- ○ Rarely
- ○ Occasionally
- ○ A moderate amount
- ○ A great deal

What is your gender?

- ○ Female
- ○ Male
- ○ Other

What is your age?

- ○ 18-24
- ○ 25-34
- ○ 35-44
- ○ 45-54
- ○ 55-64
- ○ 65-75
- ○ 75-85
- ○ 85+

What is your highest level of education?

- ◯ Primary education
- ◯ General secondary education
- ◯ Vocational education
- ◯ Higher education
- ◯ Bachelors degree
- ◯ Masters degree
- ◯ PhD degree

Below you will see a grid mapping political orientation. There are two dimensions:

(1) the horizontal axis measures whether you are <u>economically</u> left or right.
(2) the vertical axis measures whether you are <u>socially</u> libertarian or authoritarian.

Please click the position on the graphic that corresponds closest to your political orientation.

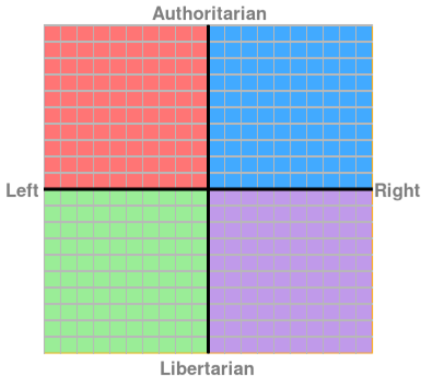

- ◯ None
- ◯ other, namely: ____

Nearly done. Thanks so much. One last thing:

As part of this study, we aim to link the survey responses you provide to what is going on in your general area. For this we would like you to share your ZIP code. If this is okay, know that we will not be able to pinpoint your exact location, but only the general area. This will help us to combine responses from multiple people in a given region, to get a more general idea of what people are thinking and feeling in that region. We naturally understand if you do not want to have your ZIP code recorded - you can decline and it will have no consequences for your current and future participation in this study.

Are you okay with recording your ZIP code?

○ Yes, I am okay with that. My Zip code is:
____

○ No, I am not okay with that.

This is a multiple-week (long-term) research study. Through your continued support, we can more precisely understand the interplay between human psychology and the challenges we are currently facing.

If you agree to be contacted again, in about a week, please provide your e-mail address. This does not obligate you to participate again; you can decide then.

○ Yes, I agree to be recontacted for another study. My e-mail is:
____

○ No, I do not want to be recontacted for another study at this time - but I do want to receive important updates and findings. My e-mail is:
____

○ No thanks, I do not want to be recontacted.

That's it for now. Thank you for your generosity in participating!

Debriefing:

The goal of this university-based psychological study is to examine how the public feels and thinks about the coronavirus epidemic and the economic impact stemming from it. Your responses will help us learn how to better predict the progression of the epidemic. The general idea is that people's mindsets, attitudes, feelings, beliefs, or intuitions, which translate into behavior, influence and predict the development of the epidemic in certain areas. The study also includes several questions about society and its challenges; as you probably saw, some questions ask about sensitive social issues. All of these questions share a singular aim: to improve the prediction of viral outbreaks and to do so as fast and accurately as possible.

If you are interested in understanding how your data is managed have a look here: Data handling.

If you have any questions or concerns about the study or your participation, you are welcome to contact the research team (psycorona@rug.nl) or have a closer look at the webpage psycorona.org. You are also welcome to contact our university ethics board at ecp@rug.nl or irbnyuad@nyu.edu referring to the following study code: PSY-1920-S-0390 or HRPP-2020-42. The principal investigators are Dr. N. P. Leander and Dr. J. J. Bélanger, New York University Abu Dhabi. If you have any questions about your rights, do not hesitate to contact privacy@rug.nl or the data protection officer at a.r.deenen@rug.nl (A.R. Deenen).

If you indicated that you would like to be recontacted, we will contact you through your e-mail. Do you have anything you wish to say for now? Please share your thoughts, advice, and feedback.

____

____

____

____

____

Your response has been recorded. Thank you for joining the effort.

If you have friends or family members around the world that you think might also want to help, please use this survey link to invite them: http://tiny.cc/corona_survey

You can also use the share buttons or make use of this little note:

Hey, I did this survey earlier and thought you might like it. It's a global survey that seeks to identify the psychological and cultural factors that can thwart the spread of the virus. The more of us that take part in the survey the better chance we can collectively make a difference. You can check it out at: http://tiny.cc/corona_survey

Share on Facebook

Share on Twitter

Share by E-mail
