# Supplementary material for: The power of the family in times of pandemic: Cross-country evidence from 93 countries
Source: SSM Popul Health. 2024 Jul 4;27:101698. doi: 10.1016/j.ssmph.2024.101698 (PMC11320607; doi:10.1016/j.ssmph.2024.101698)
Supplement: Multimedia component 2 [file mmc2.pdf]

English: **Before starting the survey please keep in mind that you can change the language in the upper right corner. Please click on the arrow below to start the survey.**

Deutsch: **Bevor Sie die Umfrage starten, beachten Sie, dass Sie die Sprache oben rechts im Browserfenster ändern können. Bitte klicken Sie unten auf den Pfeil um den Fragebogen zu beginnen.**

Nederlands: **Houd er voor het starten van de vragenlijst rekening mee dat u de taal in de rechterbovenhoek kunt wijzigen. Klik op de pijl hieronder om met de vragenlijst te beginnen.**

Italiano: **Prima di iniziare il sondaggio, tieni presente che puoi cambiare la lingua nell'angolo in alto a destra. Clicca sulla freccia in basso per iniziare il questionario.**

Français: **Avant de commençer l'enquête, veuillez noter que vous pouvez changer la langue dans le coin supérieur droit. Veuillez cliquer sur la flèche ci-dessous pour commencer l'enquête.**

Español: **Antes de comenzar la encuesta tenga en cuenta que puede cambiar el idioma en la esquina superior derecha. Por favor haga click en la flecha de abajo para empezar la encuesta.**

عربي: **الموجود السهم على اضغط فضلك من .العليا اليمنى الزاوية في اللّغة تغيير بإمكانك أنّه بالعلم الأخذ الرجاء ،الاستبيان بدء قبل**
.الاستبيان لبدء أدناه

فارسي: **عوض صفحه راست سمت بالای دکمهٔ روی بر کردن کلیک با را پرسشنامه زبانِ میتوانید شما که باشید داشته خاطر به**
.کنید کلیک صفحه پأیین فلش روی لطفا ،کردن شروع برای کنید

Bahasa Malaysia: Sebelum memulakan soal selidik, diingatkan bahawa anda boleh menukar bahasa di bahagian sudut kanan atas. **Klik pada anak panah di bawah untuk memulakan soal-selidik.**

Bahasa Indonesia: **Sebelum survei ini dimulai, Anda bisa mengubah Bahasa yang digunakan dalam survei ini di pojok kanan atas. Silahkan klik tanda panah lanjut untuk memulai survei.**

Türkçe: **Çalışmaya başlamadan önce anket dilini sağ üst köşeden Türkçe olarak seçebilirsiniz. Ankete başlamak için lütfen aşağıdaki ok işaretine tıklayınız.**

Polski: **Przed rozpoczęciem ankiety pamiętaj, że możesz zmienić język w prawym górnym rogu. Kliknij strzałkę poniżej, aby rozpocząć ankietę.**

Українська: **Перш ніж розпочати опитування, пам’ятайте, що Ви можете змінити мову у верхньому правому куті. Натисніть знизу на стрілку, щоб розпочати**

опитування.

<u>Русский:</u> Перед началом опроса имейте в виду, что язык можно изменить в правом верхнем углу. Нажмите на стрелку внизу, чтобы начать опрос.

<u>Ελληνικά:</u> Πριν ξεκινήσετε, λάβετε υπόψη ότι μπορείτε να αλλάξετε τη γλώσσα στην επάνω δεξιά γωνία. Παρακαλούμε πατήστε στο βέλος που θα βρείτε παρακάτω για να ξεκινήσετε την έρευνα.

<u>ภาษาไทย</u>**: ก่อนเริ่มการสำรวจ กรุณาระลึกไว้ว่าคุณสามารถเปลี่ยนภาษาได้ที่บริเวณมุมขวาบน กรุณาคลิ๊กลูกศรด้านล่างเพื่อเริ่มการสำรวจ**

<u>日本人:</u> この調査を始める前に、ページの右上から言語を変更することができます。 下の矢印をクリックして調査を開始してください。

<u>한국어:</u> 설문 조사를 시작하기 전에 오른쪽 상단에서 언어를 변경할 수 있습니다. 설문을 시작하시려면 아래 화살표를 눌러주시기 바랍니다.

<u>中文:</u> 開始進行調查之前，請記住您可以在右上角更改語言 **請點擊下面的箭咀開始問卷調**查。

<u>中文:</u> 在开始进行调查之前，您可以在右上角更改语言选项 请点击下面的箭头开始问卷调查。

<u>Српски:</u> Пре почетка истраживања имајте на уму да можете да промените језик у горњем десном углу. Кликните на стрелицу испод да бисте почели са истраживањем.

<u>Hrvatski:</u> Prije nego pristupite istraživanju imajte na umu da možete promijeniti jezik u gornjem desnom kutu. Da biste započeli s istraživanjem, molimo Vas da pritisnete strelicu na dnu stranice.

<u>Română:</u> Înainte de a începe să participați la studiu, vă rugăm să țineți minte faptul că puteți oricând schimba limba din colțul din dreapta sus a ecranului. Vă rugăm să dați click pe săgeata de mai jos pentru a începe studiul.

<u>Português(esa):</u> Antes de iniciar a pesquisa, lembre-se de que você pode alterar o idioma no canto superior direito. Por favor carregue na seta em baixo para comecar a pesquisa.

<u>हिन्दी:</u> सर्वे शुरू करने से पहले कृपया ध्यान रखें कि आप ऊपरी दाएं कोने में भाषा बदल सकते हैं। सर्वे शुरू करने के लिए कृपया नीचे दिए गए तीर पर क्लिक करें।

<u>Magyar:</u> Mielőtt elkezdi kitölteni a kérdőívet ne felejtse el a nyelvet magyarra állítani a jobb felső sarokban! Kérjük, kattintson a nyílra a kérdőív megkezdéséhez.

Informed consent Psycorona study

Principal Investigators: Dr. N. P. Leander, University of Groningen & Dr. J. J. Bélanger, New York University Abu Dhabi

Ethics approval: ecp@rug.nl (study code: PSY-1920-S-0390); irbnyuad@nyu.edu (study code: HRPP-2020-42)

Purpose of the research

Thank you, for volunteering, once again, to help us understand how people deal with the current situation. This survey will ask about your thoughts, feelings, and motivations regarding recent developments in society, the coronavirus, and your current economic situation. **This survey typically takes 10 minutes. It is shorter than the prior version, but many of the questions may be familiar. We plan to run these studies weekly. You can decide every week whether you want to continue getting a weekly questionnaire.**

You are under no obligation to participate in this survey. If you agree to participate, you are free to stop your participation at any time by closing the browser window. Your data will then be removed from the data set.

We are unable to provide financial reimbursement; thank you for volunteering.

Types of data collected

This is a list of sensitive questions you will encounter in this survey. Note that you can always skip any question you do not feel comfortable answering. If you are interested in understanding how we protect your privacy, personal data, and rights have a look here.

Personal data (optionally provided by you)

Directly identifiable personal data:

- Email address, location data (i.e., postal code)

Indirectly identifiable personal data:

- Gender, age range, education level, employment status, country of residence

Sensitive personal data (a.k.a. special categories of personal data)

- Political beliefs, religious beliefs, health data

How will data be collected and handled

We have specified a full list of steps on how we handle your data and protect your privacy. Especially your directly identifiable data is protected through a rigorous mechanism. If you want to see the specific steps of how we handle your data, have a look here.

Please note that developmental research studies are never completely anonymous. We will need to collect a means of contacting interested participants for follow-up surveys and will generate a random participant identification number in order to connect data over multiple measurements.

However, we have taken the following steps to protect your privacy:

(1) If you choose to be invited to follow-up surveys we will ask you to provide an email address.

Your email address and location data (postal code) are considered “directly identifiable personal data”. We will delete your email address and postal code within three months of the project completion or by January 1st, 2021 (if the study extends beyond ethical approval (i.e., January 1st, 2021), we will extend it at the consent of the ethics board; this coming fall 2020, we will do a data protection impact assessment to determine if the information is still up to date and we will take more measures if needed; we will make all that information available on our website). Your email address will also only be used for recontact and data merging. We will remove your email from the data sets after the data provided in different surveys have been linked. The data set is then effectively pseudonymized.

(2) We also ask you to optionally provide “sensitive personal data” (i.e., your religious and political beliefs, and health information; also see GDPR) as well as some “indirectly identifiable personal data” (i.e., your gender, mother language, education level, and country of residence). We collect these data in order to learn more about individual and group differences in dealing with the corona virus around the world. However, to minimize access to your sensitive and personal data the joined controller group has put a proposal system in place where access to the data is only granted upon vetted requests and only to variables absolutely necessary to the research question of collaborating researchers (see here for the full process).

(3) The data from this study will be stored in a secure location in the Department of Psychology at the Universities pursuant to data management policies of the universities and regulations of the federal government and the American Psychological Association and the Netherlands Ethics Code (Nethics code).

(4) Only members of the research team will have access to the surveydata.

(5) You have the right to obtain a copy of your data, correct it or ask to delete it until January 1st, 2021.

(6) If you would like to know more about how exactly your data will be processed and shared with collaborators see our data handling documentation and our Frequently Asked Questions section on our website. The data will be used solely for scientific and public information purposes, but will not be used for commercial purposes.

Rights of participants

If you have any questions about your rights, do not hesitate to contact privacy@rug.nl or the data protection officer at a.r.deenen@rug.nl (A.R. Deenen).

Information about storage and re-use of data

We have developed a full data management procedure about where your data is stored and how long it is stored. There you can also have a look at how we share your data with researchers from other universities.

I have read the above information. I agree to participate in this study and to the processing of my personal data as described above. I also agree to my data being transferred outside of the European Economic Area. I understand that my participation is entirely voluntary and that I may withdraw at any time by closing the browser window. I know that I can ask to have my data accessed, changed, or erased.

Do you agree to participate in this study (and confirm that you are older than 18)?

- ○ Yes, I agree to participate.

This is a multiple-week (long-term) research study. Through your continued support, we can better understand the link between human psychology and the current challenges.

If you do not want to be recontacted again, please click the unsubscribe link in the email that you received from us. You can decide every week whether you want to unsubscribe or continue receiving weekly invitations.

We will start by asking some general questions about yourself.

How did you feel over the last week?

	Very slightly or not at all	A little	Moderately	Quite a bit	Extremely
Anxious	○	○	○	○	○
Calm	○	○	○	○	○
Depressed	○	○	○	○	○
Energetic	○	○	○	○	○
Nervous	○	○	○	○	○
Exhausted	○	○	○	○	○
Inspired	○	○	○	○	○
Relaxed	○	○	○	○	○
Angry	○	○	○	○	○
Loved	○	○	○	○	○

How likely is it that the following will happen to you in the next few months?

	Exceptionally unlikely	Very Unlikely	Unlikely	About equally likely	Likely	Very likely	All but certain	Already happened
You will get infected with coronavirus.	○	○	○	○	○	○	○	○
Your personal situation will get worse due to economic consequences of coronavirus.	○	○	○	○	○	○	○	○

Which of the following categories best describes your employment status <u>during the last week</u> (multiple may apply)?

☐ Employed, working 1-23 hours per week

☐ Employed, working 24-39 hours per week

☐ Employed, working 40 or more hours per week

☐ Self-employed

☐ Not employed, looking for work

☐ Not employed, **not** looking for work

☐ Lost my employment in the last week

☐ Lost my employment in the last week as my job has become temporarily not available (e.g., restaurant worker, driver, etc.)

☐ Homemaker

☐ Retired

☐ Disabled, not able to work

☐ Student

☐ Volunteering

☐ Other ____

In the last week, how many hours did you work?

	0	8	16	24	32	40	48	56	64	72	80
Hours worked											

Agree or disagree:

	Strongly disagree	Disagree	Neither agree nor disagree	Agree	Strongly agree
I fear that things will go wrong in society.	○	○	○	○	○
Chances are, I will soon lose my job.	○	○	○	○	○
I am financially strained.	○	○	○	○	○
Not a lot is done for people like me in the country I live in.	○	○	○	○	○

In the past 7 days, how much social contact have you had with <u>people who live outside your household</u>?

	In the past 7 days, how many days did you have **in-person** (face-to-face) contact with ...								In the past 7 days, how many days did you have **online** (video or voice) contact with ...							
	0	1	2	3	4	5	6	7	0	1	2	3	4	5	6	7
...friends or relatives																
...other people in general																

In the last week, how satisfied were you with your personal relationships?

- ○ 1 Extremely unsatisfied
- ○ 2
- ○ 3
- ○ 4
- ○ 5
- ○ 6
- ○ 7
- ○ 8
- ○ 9
- ○ 10 Extremely satisfied

The following questions are about how you felt during the past week.

During the past week, did you...

	Never	Rarely	Sometimes	Often	All the time
...feel **lonely**?	○	○	○	○	○

How is your current <u>mental</u> health?

- ○ 1: Terrible
- ○ 2
- ○ 3
- ○ 4
- ○ 5
- ○ 6
- ○ 7
- ○ 8
- ○ 9
- ○ 10: Excellent

In general, how happy would you say you are?

- ○ 1 Extremely unhappy
- ○ 2
- ○ 3
- ○ 4
- ○ 5
- ○ 6
- ○ 7
- ○ 8
- ○ 9
- ○ 10 Extremely happy

In general, how satisfied are you with your life?

- ○ Very dissatisfied
- ○ Dissatisfied
- ○ Slightly dissatisfied
- ○ Slightly satisfied
- ○ Satisfied
- ○ Very satisfied

In general, how much do you trust each of the following to take the right measures to deal with the coronavirus pandemic?

	Not at all	A little	A moderate amount	A lot	A great deal
The government of your country	○	○	○	○	○
Your community	○	○	○	○	○

Agree or disagree:

	Strongly disagree	Disagree	Somewhat disagree	Neither agree nor disagree	Somewhat agree	Agree	Strongly agree
I have high hopes that the situation regarding coronavirus will improve.	○	○	○	○	○	○	○
I think that the country I live in is able to fight the Coronavirus.	○	○	○	○	○	○	○

Agree or disagree:

"I am willing to..."

	Strongly disagree	Disagree	Somewhat disagree	Neither agree nor disagree	Somewhat agree	Agree	Strongly agree
...help others who suffer from coronavirus.	○	○	○	○	○	○	○
...protect vulnerable groups from coronavirus even at my own expense.	○	○	○	○	○	○	○
...make personal sacrifices to prevent the spread of coronavirus.	○	○	○	○	○	○	○

Agree or disagree:

"To minimize my chances of getting coronavirus, I..."

	Strongly disagree	Disagree	Somewhat disagree	Neither agree nor disagree	Somewhat agree	Agree	Strongly agree
...wash my hands more often.	○	○	○	○	○	○	○
...avoid crowded spaces.	○	○	○	○	○	○	○
...put myself in quarantine.	○	○	○	○	○	○	○

Agree or disagree:

"I would sign a petition that supports..."

	Strongly disagree	Disagree	Somewhat disagree	Neither agree nor disagree	Somewhat agree	Agree	Strongly agree
...mandatory vaccination once a vaccine has been developed for coronavirus.	○	○	○	○	○	○	○
...mandatory quarantine for those that have coronavirus and those that have been exposed to the virus.	○	○	○	○	○	○	○

Do you personally know anyone who <u>currently</u> has coronavirus?
(click all that apply)

- ☐ Yes, myself
- ☐ Yes, a member of my family
- ☐ Yes, a close friend
- ☐ Yes, someone I know
- ☐ Yes, someone else
- ☐ No, I do not know anyone

To what extent is your community….

	1: Not at all	2	3	4	5	6: Very much
...punishing people who deviate from the rules that have been put in place in response to the Coronavirus?	◯	◯	◯	◯	◯	◯

To what extent….

	1: Messages are completely unclear/ ambiguous	2	3	4	5	6: Messages are very clear/ unambiguous
...are you getting clear, unambiguous messages about what to do about the Coronavirus?	◯	◯	◯	◯	◯	◯

The next questions are about your experiences with the economic and financial consequences of coronavirus.

How knowledgeable are you about the potential economic and financial consequences of coronavirus in this country?

◯ Not at all knowledgeable

◯ Slightly knowledgeable

◯ Moderately knowledgeable

◯ Very knowledgeable

◯ Extremely knowledgeable

Agree or disagree:

	Strongly disagree	Disagree	Somewhat disagree	Neither agree nor disagree	Somewhat agree	Agree	Strongly agree
I have high hopes that the situation regarding the economic and financial consequences of coronavirus will improve.	○	○	○	○	○	○	○
I think that this country is able to fight the economic and financial consequences of coronavirus.	○	○	○	○	○	○	○

Indicate your agreement or disagreement with the following statements.

To help with the economic and financial consequences of coronavirus, I am willing to...

	Strongly disagree	Disagree	Somewhat disagree	Neither agree nor disagree	Somewhat agree	Agree	Strongly agree
...help others who suffer from such consequences.	○	○	○	○	○	○	○
...protect vulnerable groups from such consequences, even at my own expense.	○	○	○	○	○	○	○

Indicate your agreement or disagreement with the following statements.

If it would alleviate the economic and financial consequences of coronavirus, I would sign a petition that supports...

	Strongly disagree	Disagree	Somewhat disagree	Neither agree nor disagree	Somewhat agree	Agree	Strongly agree
...giving the government more authority over people.	○	○	○	○	○	○	○
...increased government spending.	○	○	○	○	○	○	○

When dealing with stressful situations, what do you usually do?

	(Almost) never	Rarely	Sometimes	Often	(Almost) always
I try to come up with a strategy about what to do.	○	○	○	○	○
I distract myself to avoid thinking about the subject.	○	○	○	○	○

The next questions are about your overall experiences.

In the past week, how often did you leave your home?

○ I did not leave my home.

○ Once or twice.

○ Three times.

○ Four times or more.

In the previous question, you have indicated that in the past week, you left the home at least once.
What were the reasons for leaving your home?
(Select all that apply)

- ☐ I had to go to work.
- ☐ I had errands to run.
- ☐ Doing something for another person (e.g. helping with groceries)
- ☐ For leisure purposes with others (e.g., meeting up with friends, seeing family, going to the cinema, etc.)
- ☐ For leisure purposes alone (e.g., running, going for a walk, etc.)
- ☐ Other, please specify: ____

In the past week, did you stock up on extra food or other supplies when running errands or shopping for groceries?

- ○ Not at all
- ○ A little more than normal
- ○ A moderate amount more than normal
- ○ A lot more than normal
- ○ A great deal more than normal

Indicate your agreement or disagreement with the following statements.

	Strongly disagree	Disagree	Somewhat disagree	Neither agree nor disagree	Somewhat agree	Agree	Strongly agree
Time is moving very slowly.	○	○	○	○	○	○	○

Agree or disagree:

	Strongly disagree	Disagree	Somewhat disagree	Neither agree nor disagree	Somewhat agree	Agree	Strongly agree
I replay memories of the past in my mind.	○	○	○	○	○	○	○
I focus on what is currently happening in my life.	○	○	○	○	○	○	○
I think about what my future has in store.	○	○	○	○	○	○	○

In the past 7 days,...

	Never	Sometimes	About half the time	Most of the time	Always
How often did you occupy your time with activities you **want** to do?	○	○	○	○	○
How often did you occupy your time with activities you **have** to do?	○	○	○	○	○

In the absence of an effective medical treatment or a vaccine, social distancing measures are the most effective means of controlling the pandemic.

○ Strongly disagree

○ Somewhat disagree

○ Neither agree nor disagree

○ Somewhat agree

○ Strongly agree

How likely are you to get vaccinated against coronavirus once a vaccine becomes available?

- ◯ Extremely unlikely
- ◯ Unlikely
- ◯ Undecided
- ◯ Likely
- ◯ Extremely likely

Agree or disagree:

	Strongly disagree	Disagree	Somewhat disagree	Neither agree nor disagree	Somewhat agree	Agree	Strongly agree
The country I live in is doing a better job in fighting the coronavirus than most other countries.	◯	◯	◯	◯	◯	◯	◯
I feel as sense of solidarity with people in my country.	◯	◯	◯	◯	◯	◯	◯
I feel a sense of solidarity with people in other countries.	◯	◯	◯	◯	◯	◯	◯

Nearly done. Thanks so much. One last thing:

As part of this study, we aim to link the survey responses you provide to what is going on in your general area. For this we would like you to share your current ZIP or postal code. If this is okay, know that we will not be able to pinpoint your exact location, but only the general area. This will help us to combine responses from multiple people in a given region, to get a more general idea of what people are thinking and feeling in that region. We naturally understand if you do not want to have your ZIP or postal code recorded - you can decline and it will have no consequences for your current and future participation in this study.

Are you okay with recording your ZIP or postal code?

- ◯ Yes, I am okay with that. My Zip code is: ____

- ◯ No, I am not okay with that.

That's it for now. Thank you for your generosity in participating!

Debriefing:
The goal of this university-based psychological study is to examine how the public feels and thinks about the coronavirus epidemic and the economic impact stemming from it. Your responses will help us learn how to better predict the progression of the epidemic. The general idea is that people's mindsets, attitudes, feelings, beliefs, or intuitions, which translate into behavior, influence and predict the development of the epidemic in certain areas. The study also includes several questions about society and its challenges; as you probably saw, some questions ask about sensitive social issues. All of these questions share a singular aim: to improve the prediction of viral outbreaks and to do so as fast and accurately as possible.
If you are interested in understanding how your data is managed have a look here: Data handling.

If you have any questions or concerns about the study or your participation, you are welcome to contact the research team (psycorona@rug.nl) or have a closer look at the webpage psycorona.org. You are also welcome to contact our university ethics board at ecp@rug.nl or irbnyuad@nyu.edu referring to the following study code: PSY-1920-S-0390 (RUG) or HRPP-2020-42 (NYU). The principal investigators are Dr. N. P. Leander, University of Groningen, and Dr. J. J. Bélanger, New York University Abu Dhabi. If you have any questions about your rights, do not hesitate to contact privacy@rug.nl or the data protection officer (A.R. Deenen) at a.r.deenen@rug.nl.

If you did not click the “unsubscribe” link in the email you received from us, we will contact you again through your email.

Do you have anything you wish to say for now? Please share your thoughts, advice, and feedback.

____
