# Supplementary material for: The power of the family in times of pandemic: Cross-country evidence from 93 countries
Source: SSM Popul Health. 2024 Jul 4;27:101698. doi: 10.1016/j.ssmph.2024.101698 (PMC11320607; doi:10.1016/j.ssmph.2024.101698)
Supplement: Multimedia component 3 [file mmc3.pdf]

Supporting Information for

The power of the family in times of pandemic: Cross-country evidence from 93 countries

This file includes:

Supporting text
Figures S1 to S6
Tables S1 to S12
SI References

Other supporting materials for this manuscript include the following:

PsyCorona Survey Sample Questionnaires (Baseline wave and Wave 4)

Supporting Information Text

Alternative construction approach of family ties

We tried an alternative construction method for the strength of family ties, in which we summed up the numeric answers to the two WVS questions and then calculated the average from five waves of survey. Given family ties constructed by the principal component analysis (PCA) has slightly larger variance compared with that from the summation approach (Table S10), we chose to use the latest wave of WVS when conducting PCA so that we could capture the most recent information should there be any changes. Table S11 shows the pairwise correlation coefficients among the original survey answers and the two measures of family ties. Similar to Alesina and Giuliano's study (Alesina & Giuliano, 2010), these two family ties measures almost perfectly correlate with each other. Also, the ranking of strength of family ties by summation (Fig. S6) is similar to those presented in Fig. 1.

Historical Climate Variability

We focused on variability in temperature and precipitation for measuring weather-related risk during pre-industrial times. Building upon the study of Buggle and Durante (*46*), our measure of climate variability is the average variability during the growing seasons, spring and summer, spanning the period from 1500 to 1750. The data was obtained from the European Seasonal Temperature and Precipitation Reconstruction (ESTPR), the most recent and advanced reconstructions of European climate over the last 500 years (Luterbacher et al., 2004; Pauling et al., 2006). Each cell in the ESTPR grid has a width of 0.5 degree, which corresponds to approximately 56 kilometers at the equator. Cell-level data includes seasonal observations between 1500 and 2000.

We followed Buggle and Durante (*46*) in construction of climate variability measure. We used x_{isy} to denote the value of a climatic variable x in cell i in season s in the year y. Our procedure began with the calculation of the season-specific variability for variable x in cell i, denoted by σ_{is}, through the computation of the standard deviation of x_{isy} across all years between 1500 to 1750. Then, for each cell i, we averaged σ_{is} for growing seasons to obtain σ_i. Finally, we averaged σ_i over all cells within country c to obtain a country-level measure of variability in growing seasons, that is, σ_c.

Moreover, we used PCA to construct a composite indicator of climatic variability. This principal component reflects the overall country-level climate variability in the pre-industrial era.

Country-level vaccination coverage

The vaccination coverage data was sourced from Our World in Data COVID-19 vaccination dataset. Our World in Data is a project affiliated with the University of Oxford that has aggregated COVID-19 data from government sources *(33)*. It encompasses data from December 13, 2020, which marks the initial release of vaccination, and has been consistently updated thereafter. In instances where data on a specific day of the survey is missing for a country, we substituted the nearest available non-missing data prior to that particular PsyCorona survey wave.

Sample Attrition of PsyCorona survey

To assess sample attrition in PsyCorona survey, we divided respondents into "discontinued" and "continued" subsamples. Respondents who have participated in at least one wave after Baseline survey are in the "continued" subsample. And those who participated in the baseline survey but not in any of the subsequent waves are in the "discontinued" subsample. Additionally, because question regarding willingness to receive vaccination was introduced in a latter wave, individuals answered this question are in the "continued" subsample automatically. Table S12 indicates that attrition did not happen randomly, which may lead to attrition bias.

Weighting

To ameliorate concerns for non-nationally representative sample and sample attrition, we weighed our sample by three types of weights: demographic weight, population scaled weight, and attrition weight.

First, we employed the post-stratification method for demographic weighting. We segmented the population into three age-brackets: 18-34, 35-64, and 65+. Subsequently, we computed a weighting matrix to ensure that the sex-age distribution within each country mirrored their respective 2000 census data. In cases where a particular age group in a country's sample lacks respondents in the PsyCorona survey, that country is excluded from the subsequent analysis. And this is the reason why only 31 countries were involved in this weighted sample analysis.

In order to standardize the sample count across all countries after the demographic weighting process, we applied an additional weight (W_1). This weight was proportional to the demographic weight to ensure sample from each country consistently represented 1000 cases. Then, we used W_1 to construct a population scaled weight ($W_{country}$) in the following way:

$$W_{country} = \frac{W_1}{1000} \times \ Population\ of a\ country$$

$W_{country}$ is used as cross-sectional weights in the baseline wave, and it gave an N for each country equaled to the population size of the country covered by the sample.

Furthermore, to account for attrition bias, we ran logistic regression with response status as the outcome variable, and then we used the inverse of predicted probability of responding as attrition weight. We multiplied the attrition weight with $W_{country}$ as the panel weights for waves after the baseline.

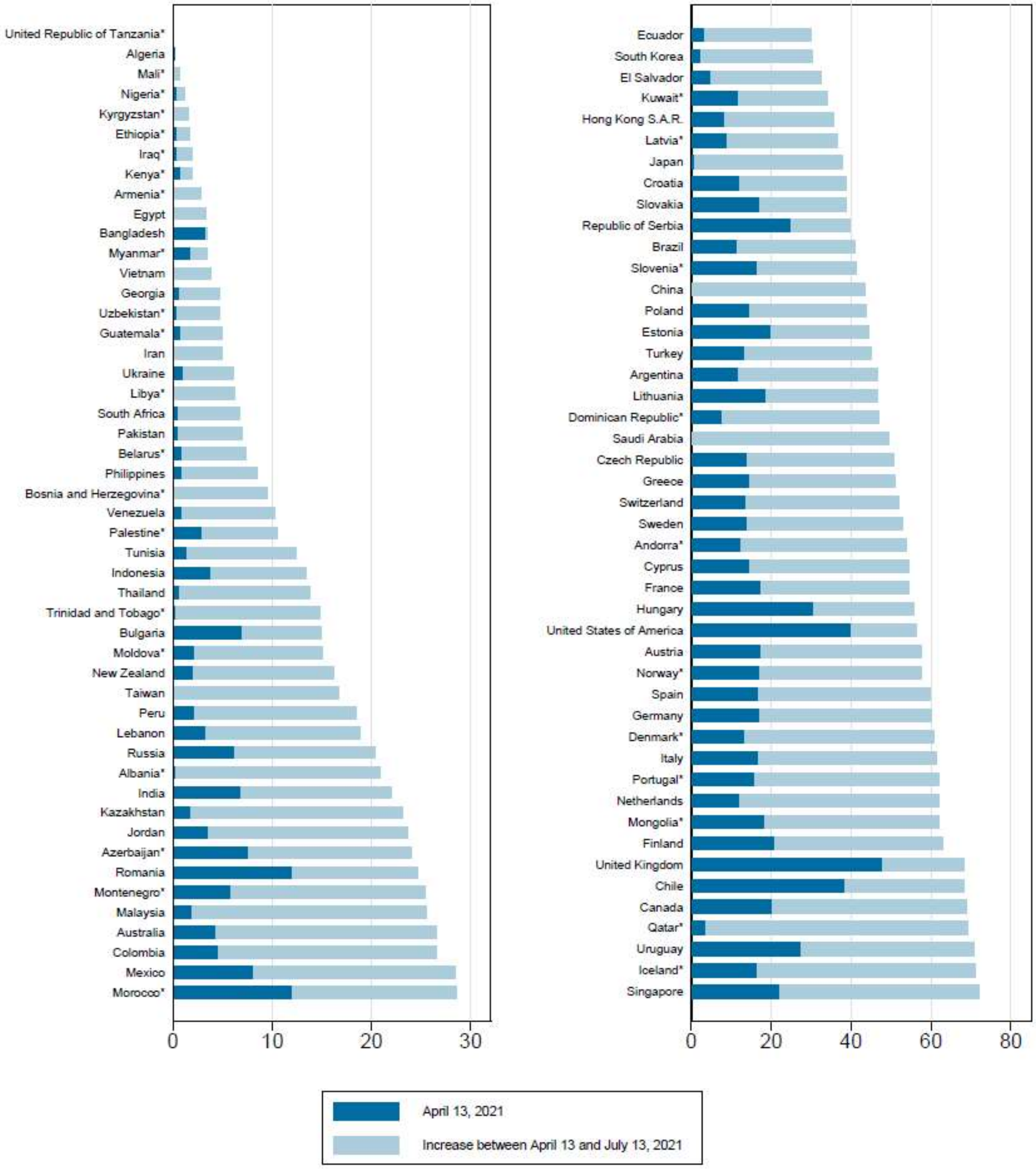

Fig. S1. Vaccination coverage by country (region) on April 13, 2021 and July 13, 2021 among 93 countries. Countries (regions) are ranked by their vaccination coverage on July 13, 2021. A star indicates there were no participants from that country in the PsyCorona survey in both survey waves on April 13 and July 13, 2021. Data were extracted from Our World in Data.

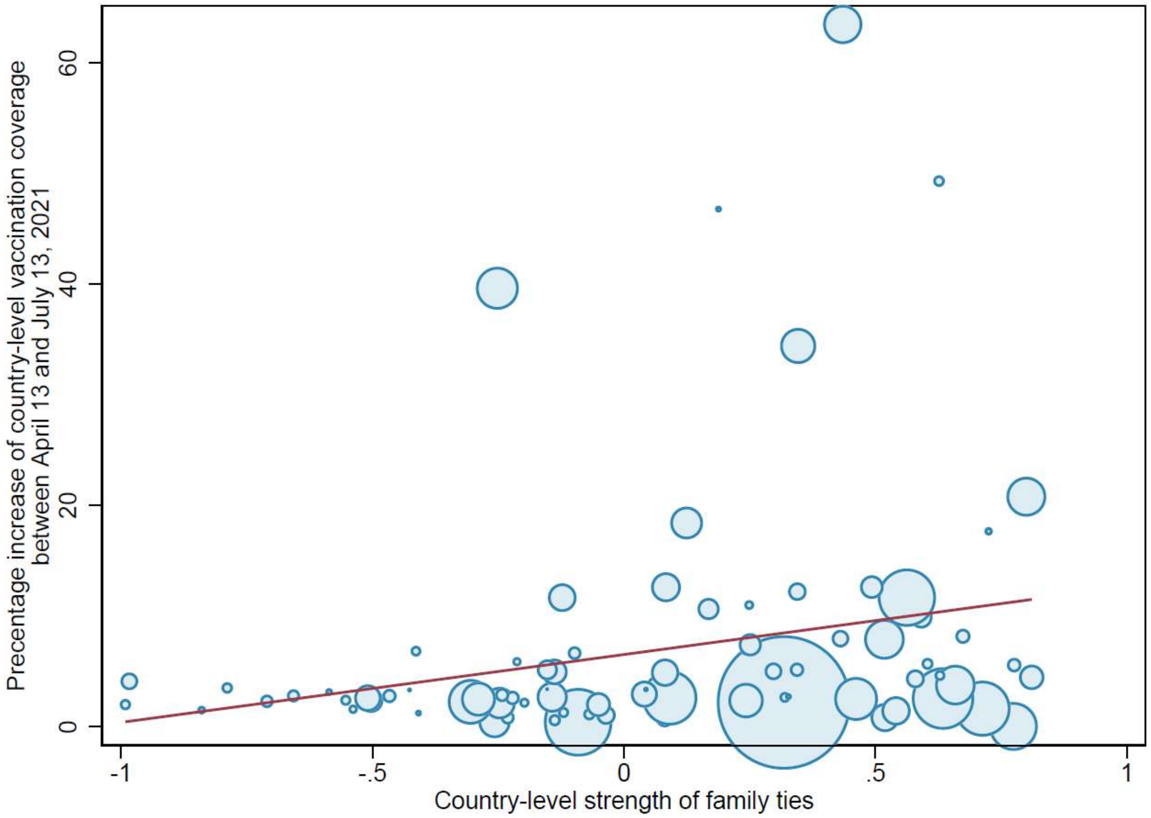

Fig. S2. Association between family ties and percentage increase of country-level vaccination coverage between April 13 and July 13, 2021 among 93 countries. Percentage increase was calculated by dividing the increase in vaccination coverage from April to July by vaccination coverage in April. The slope of the fitted line is 35.67 (P = 0.024). The size of each circle represents the population size of a given country in 2021. Six countries were excluded from the graph for the following reasons: Albania, Armenia and Libya were excluded from the graph as outliers (percentage change between 100% and 700%). Vaccination coverage data of China and Saudi Arabia did not become available until June 2021. And Tanzania's vaccination coverage was zero in both April and July 2021. Country-level vaccination coverage was extracted from Our World in Data. Country-level family ties were computed by the author through PCA approach using the WVS.

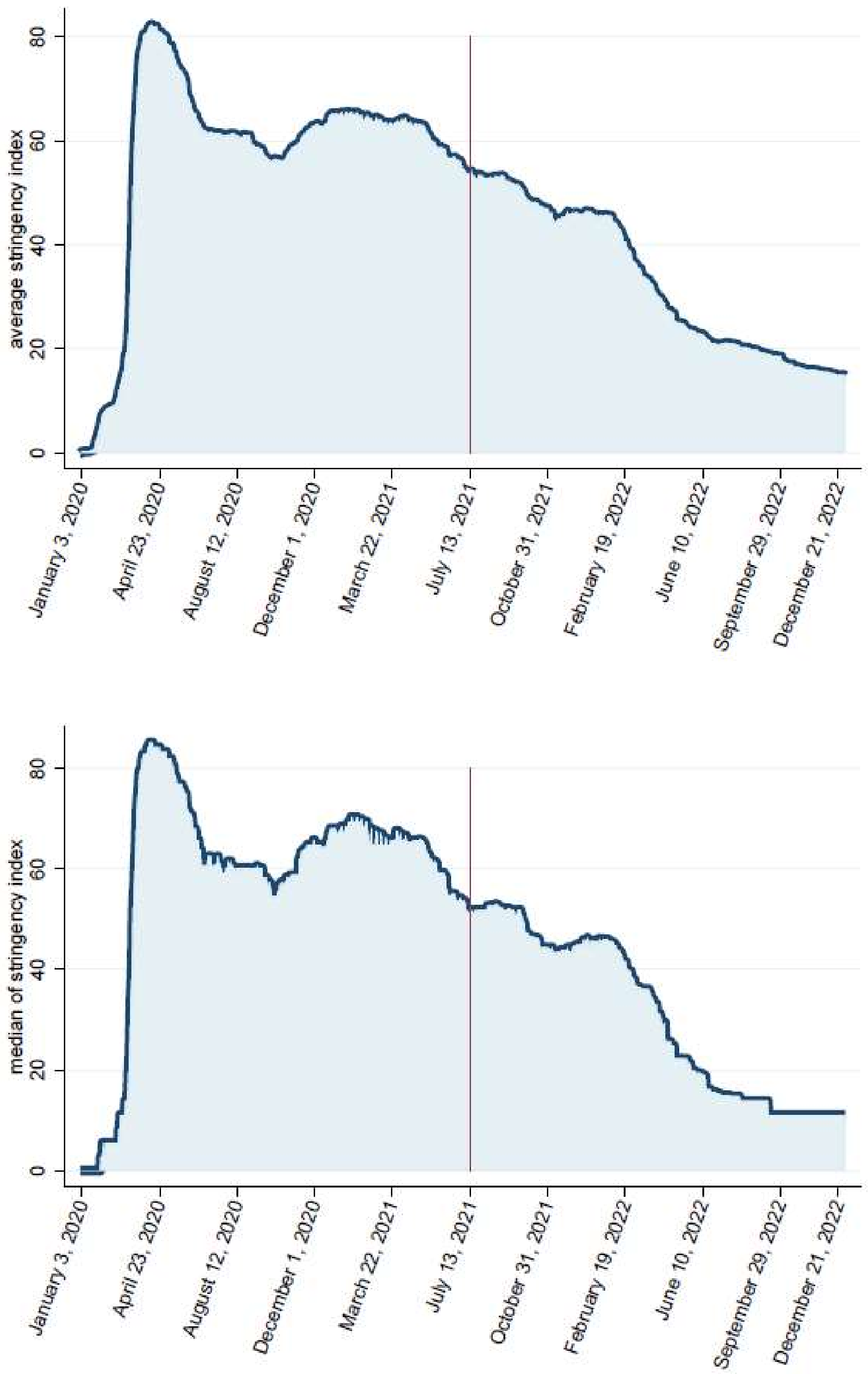

Fig. S3. Evolution of government stringency index of 93 countries. The stringency index is a composite measure based on nine response indicators including school closures, workplace closures, and travel bans, rescaled to a value from 0 to 100 (100 = strictest). The trend of the average value of the index among the 93 countries is presented in the top panel and the trend of the median value is at the bottom panel. The red vertical line marks the date of the last PsyCorona survey wave. Data was extracted from OxCGRT.

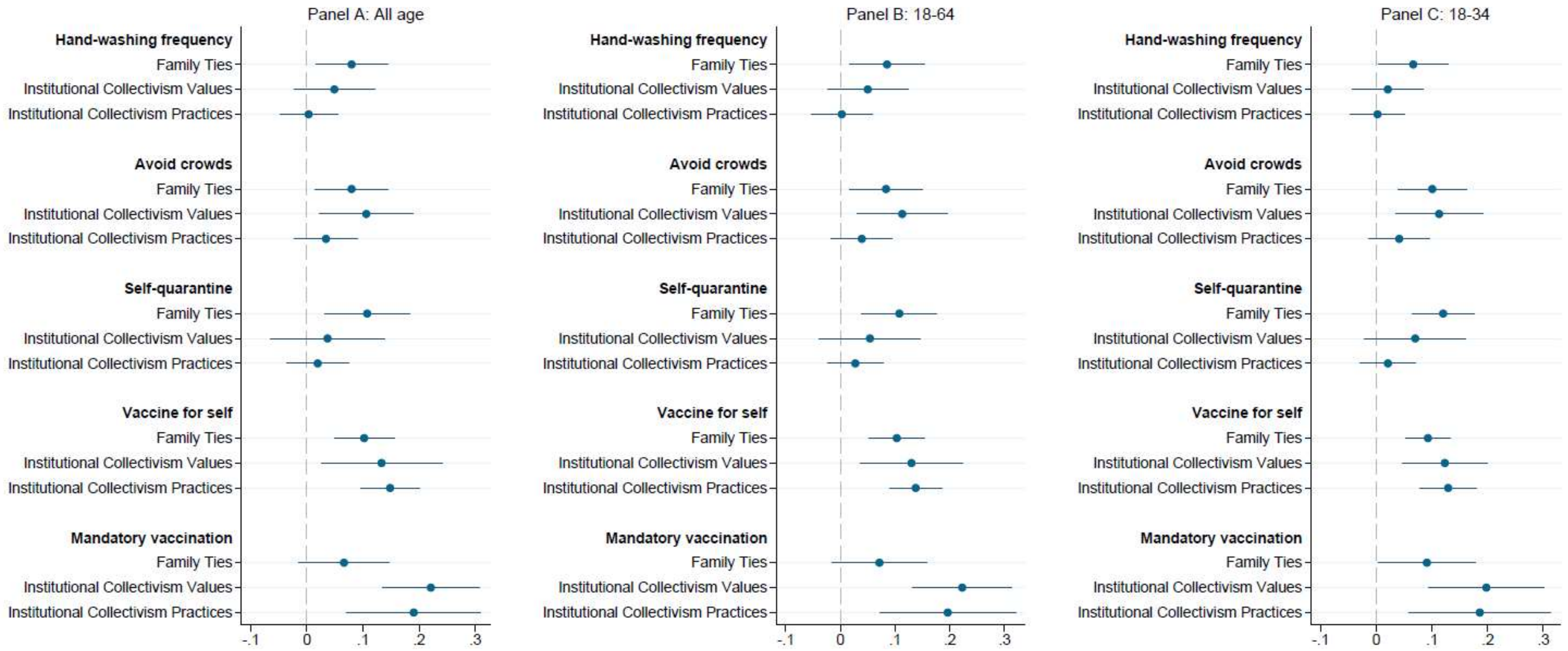

Fig. S4. Family ties vs. Institutional Collectivism. Each panel shows, for different age groups, standardized regression coefficients of family ties, institutional collectivism values and practice with 95% confidence intervals for each of the five attitude outcomes. All regressions controlled for individual covariates, country-level 7-day average daily increase of confirmed cases per capita one week prior to survey wave, country-level government stringency index and vaccination coverage on the day of the survey, natural log of GDP per capita in 2019, and out-of-pocket expenditure as percent of health expenditure in 2019. Standard errors were clustered at the country level. Numeric coefficients are also presented in Table S8.

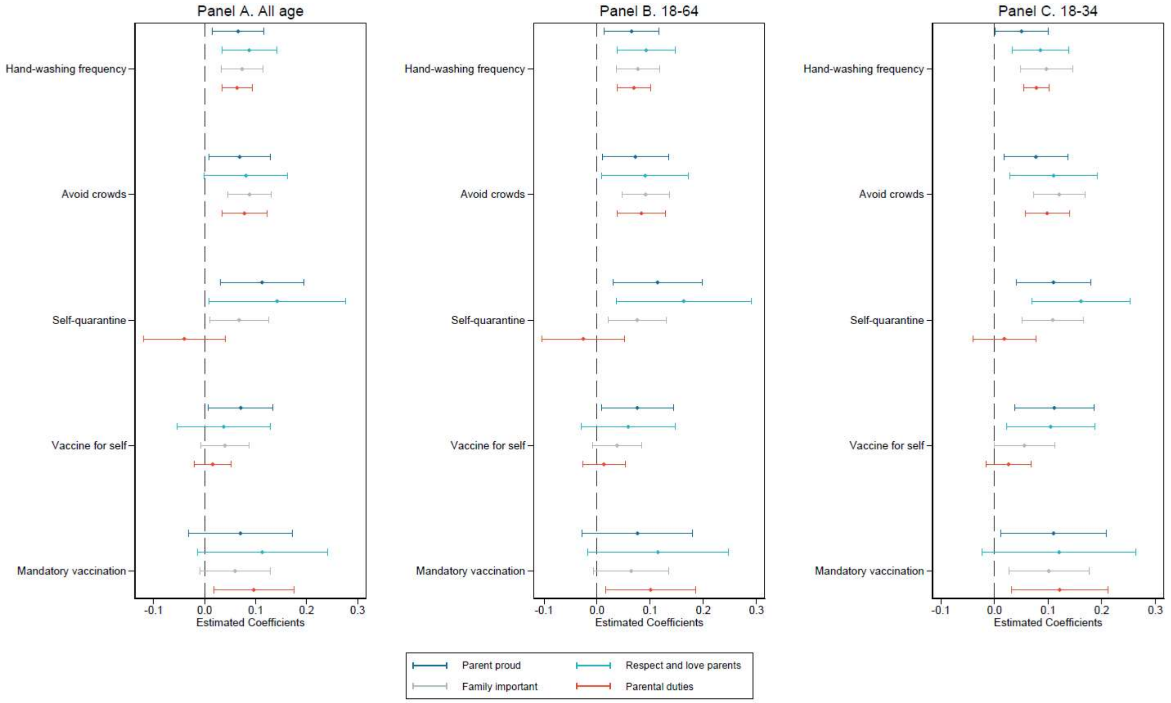

Fig. S5. Children's attitudes towards parents vs. parental attitudes towards children. Each panel shows, for different age groups, standardized regression coefficients of different WVS questions regarding family ties, with 90% confidence intervals for each of the five attitude outcomes. *Parent proud:* whether one of respondent's main goals in life had been to make her parents proud. *Respect and love parents,* which asks whether (1) regardless of what the qualities and faults of one's parents are, one must always love and respect them, and (2) one does not have the duty to respect and love parents who have not earned it. *Family important:* how important the family was in respondent's life. *Parental duties*, which asks whether: (1) It is the parents' duty to do their best for their children, even at the expense of their own well-being, and (2) parents have a life on their own. All regressions controlled for individual covariates, country-level 7-day average daily increase of confirmed cases per capita one week prior to survey wave, country-level government stringency index and vaccination coverage on the day of the survey, natural log of GDP per capita in 2019, and out-of-pocket expenditure as percent of health expenditure in 2019. Standard errors were clustered at the country level.

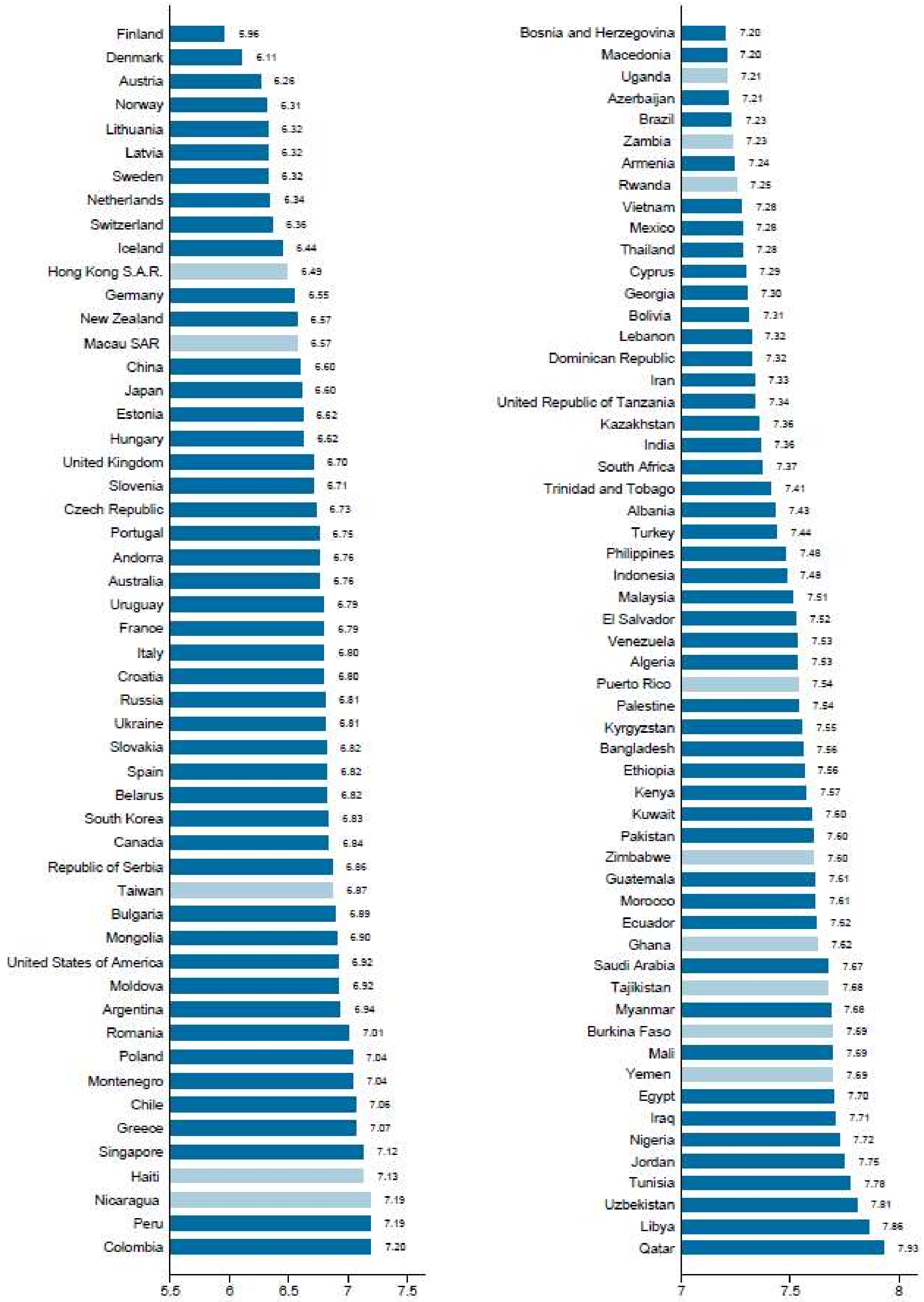

Fig. S6. Strength of family ties by country (region) constructed by summation. Higher values correspond to stronger family ties. This measure is constructed by summing the numeric responses to two questions from (at most) five most recent waves of WVS starting from 1994 and then divided by number of waves used. Light blue bars indicate countries and regions not included in our analysis because no matching data is available in PsyCorona.

Table S1. Descriptive statistics of World Values Survey 2017 wave.

	Mean	SD	Min	Max
Age	49.60	17.66	18	82
Female	55.83%	0.50	0	1
High school education and above	79.50%	0.40	0	1
Unemployed	8.52%	0.28	0	1
N=58,401				

The following notes is applicable to Table S2 – S9

Table presents standardized coefficients from random effects regression models, adjusted for individual characteristics: age, gender, education level, employment status, whether feeling financially strained, country-level attributes: 7-day average daily increase of confirmed cases per capita one week prior to survey wave, government stringency index on the day of survey wave, natural log of GDP per capita in 2019, and out-of-pocket expenditure as percent of health expenditure in 2019. Standard errors were clustered at the country level.

Country-level vaccination coverage at the time of the survey was controlled for in Table S3 – S8. Additionally, results in Table S9 controlled for 7-day average country-level vaccination coverage 30 days prior to the survey wave.
Standard errors are reported in the parentheses. *indicates significance at the 10% level, **indicates significance at the 5% level, and *** indicates significance at the 1% level.

Table S2. Baseline regression results.

	Hand-washing frequency	Avoid crowds	Self-quarantine	Vaccine for self	Mandatory vaccination
Panel A1: All age					
Family ties	0.062**	0.075**	0.106***	0.080***	0.081
	(0.030)	(0.031)	(0.036)	(0.030)	(0.054)
Family ties (weighted)	0.062**	0.074**	0.100***	0.075***	0.075
	(0.028)	(0.031)	(0.034)	(0.028)	(0.052)
N	111,526	111,526	111,526	50,025	88,329
Panel A2: 18-64					
Family ties	0.062**	0.078**	0.109***	0.084***	0.089
	(0.030)	(0.033)	(0.036)	(0.030)	(0.054)
Family ties (weighted)	0.062**	0.077**	0.102***	0.077***	0.082
	(0.028)	(0.032)	(0.034)	(0.028)	(0.052)
N	96,547	96,547	96,547	39,934	80,372
Panel A3: 18-34					
Family ties	0.044	0.083**	0.112***	0.108***	0.119**
	(0.039)	(0.033)	(0.032)	(0.036)	(0.051)
Family ties (weighted)	0.044	0.080**	0.106***	0.098***	0.112**
	(0.036)	(0.032)	(0.031)	(0.033)	(0.049)
N	41,647	41,647	41,647	12,699	39,134
Panel B1: All age					
Family ties	0.066**	0.080***	0.112***	0.080***	0.087
	(0.027)	(0.030)	(0.034)	(0.030)	(0.054)
Family ties (weighted)	0.065**	0.079***	0.106***	0.075***	0.080
	(0.026)	(0.029)	(0.032)	(0.028)	(0.052)
N	107,527	107,527	107,527	48,552	84,665
Panel B2: 18-64					
Family ties	0.064**	0.082**	0.115***	0.084***	0.094*
	(0.028)	(0.032)	(0.035)	(0.030)	(0.054)
Family ties (weighted)	0.064**	0.081***	0.108***	0.078***	0.087*
	(0.026)	(0.031)	(0.033)	(0.028)	(0.052)
N	92,927	92,927	92,927	38,645	76,963
Panel B3: 18-34					
Family ties	0.045	0.086***	0.116***	0.104***	0.125**
	(0.035)	(0.030)	(0.030)	(0.037)	(0.051)
Family ties (weighted)	0.044	0.084***	0.110***	0.094***	0.116**
	(0.032)	(0.029)	(0.029)	(0.034)	(0.049)
N	39,963	39,963	39,963	12,260	37,443

Table S3. Regression results controlling for country-level vaccination coverage.

	Hand-washing frequency	Avoid crowds	Self-quarantine	Vaccine for self	Mandatory vaccination
Panel A1: All age					
Family ties	0.061**	0.074**	0.105***	0.066**	0.079
	(0.030)	(0.031)	(0.035)	(0.031)	(0.052)
Family ties (weighted)	0.061**	0.073**	0.099***	0.061**	0.072
	(0.028)	(0.030)	(0.034)	(0.029)	(0.051)
N	111,526	111,526	111,526	50,025	88,329
Panel A2: 18-64					
Family ties	0.061**	0.077**	0.108***	0.069**	0.086
	(0.030)	(0.032)	(0.036)	(0.032)	(0.053)
Family ties (weighted)	0.061**	0.076**	0.101***	0.064**	0.079
	(0.028)	(0.031)	(0.034)	(0.030)	(0.051)
N	96,547	96,547	96,547	39,934	80,372
Panel A3: 18-34					
Family ties	0.042	0.081**	0.111***	0.100***	0.116**
	(0.039)	(0.033)	(0.031)	(0.038)	(0.050)
Family ties (weighted)	0.042	0.079**	0.105***	0.090***	0.109**
	(0.036)	(0.031)	(0.031)	(0.034)	(0.048)
N	41,647	41,647	41,647	12,699	39,134
Panel B1: All age					
Family ties	0.065**	0.079***	0.111***	0.065**	0.085
	(0.027)	(0.030)	(0.034)	(0.032)	(0.053)
Family ties (weighted)	0.064**	0.078***	0.105***	0.060**	0.077
	(0.026)	(0.029)	(0.032)	(0.029)	(0.051)
N	107,527	107,527	107,527	48,552	84,665
Panel B2: 18-64					
Family ties	0.063**	0.080***	0.114***	0.068**	0.091*
	(0.028)	(0.031)	(0.034)	(0.033)	(0.053)
Family ties (weighted)	0.062**	0.080***	0.107***	0.063**	0.084*
	(0.027)	(0.030)	(0.032)	(0.030)	(0.051)
N	92,927	92,927	92,927	38,645	76,963
Panel B3: 18-34					
Family ties	0.043	0.084***	0.114***	0.097**	0.121**
	(0.035)	(0.030)	(0.029)	(0.039)	(0.050)
Family ties (weighted)	0.043	0.082***	0.108***	0.087**	0.113**
	(0.032)	(0.029)	(0.028)	(0.035)	(0.048)
N	39,963	39,963	39,963	12,260	37,443

Table S4. Regression results with weighted sample.

Analysis restricted to 31 countries with balanced age representation in PsyCorona Survey

	Hand-washing frequency	Avoid crowds	Self-quarantine	Vaccine for self	Mandatory vaccination
Panel A1: All age					
Family ties	0.178***	0.280***	0.298***	0.199***	0.200**
	(0.051)	(0.077)	(0.064)	(0.062)	(0.074)
Family ties (weighted)	0.166***	0.263***	0.283***	0.183***	0.181**
	(0.048)	(0.072)	(0.060)	(0.060)	(0.072)
N	93,537	93,537	93,537	41,678	63,197
Population N	11,256,685,857	11,256,685,857	11,256,685,857	8,727,632,871	4,953,654,991
Panel A2: 18-64					
Family ties	0.184***	0.287***	0.308***	0.202***	0.208***
	(0.051)	(0.076)	(0.063)	(0.061)	(0.072)
Family ties (weighted)	0.172***	0.271***	0.291***	0.186***	0.189**
	(0.048)	(0.072)	(0.058)	(0.058)	(0.070)
N	81,915	81,915	81,915	34,081	57,378
Population N	10,262,902,269	10,262,902,269	10,262,902,269	7,919,665,744	4,591,083,519
Panel A3: 18-34					
Family ties	0.183***	0.315***	0.289***	0.215***	0.286***
	(0.065)	(0.077)	(0.062)	(0.075)	(0.076)
Family ties (weighted)	0.168**	0.290***	0.269***	0.186**	0.257***
	(0.062)	(0.075)	(0.058)	(0.075)	(0.076)
N	35,007	35,007	35,007	10,632	27,823
Population N	5,006,341,610	5,006,341,610	5,006,341,610	3,706,699,208	2,403,503,214

Table S5. Regression results using family ties constructed by WVS 1995-2009.

Family ties measure in Panel A was constructed by using WVS 2005-2009. If a country did not participate in WVS 2005-2009, we used its most recent wave available prior to 2005. In Panel B, we conducted PCA first using each of the three waves of WVS between 1995 and 2009, and then used the average value across these three waves as family ties measure in regression analysis.

	Hand-washing frequency	Avoid crowds	Self-quarantine	Vaccine for self	Mandatory vaccination
Panel A: Latest: Wave 5 (2005-2009) - 52 countries					
Panel A1: All age					
Family ties	0.059*	0.075**	0.094**	0.053*	0.093***
	(0.030)	(0.030)	(0.046)	(0.027)	(0.035)
Family ties (weighted)	0.060*	0.077***	0.102**	0.057**	0.097***
	(0.032)	(0.029)	(0.042)	(0.026)	(0.034)
N	92,229	92,229	92,229	43,196	75,115
Panel A2: 18-64					
Family ties	0.061**	0.084***	0.099**	0.067**	0.102***
	(0.031)	(0.030)	(0.042)	(0.030)	(0.034)
Family ties (weighted)	0.062*	0.085***	0.106***	0.071**	0.105***
	(0.032)	(0.028)	(0.038)	(0.029)	(0.034)
N	79,877	79,877	79,877	33,329	67,859
Panel A3: 18-34					
Family ties	0.042	0.084***	0.094***	0.099***	0.122***
	(0.036)	(0.031)	(0.036)	(0.035)	(0.029)
Family ties (weighted)	0.041	0.085***	0.098***	0.102***	0.124***
	(0.037)	(0.030)	(0.034)	(0.032)	(0.028)
N	33,853	33,853	33,853	10,387	32,994
Panel B: Average of: Wave 3 (1995-1998) – Wave 5 (2005-2009) - 77 countries					
Panel B1: All age					
Family ties	0.054*	0.068***	0.080**	0.074**	0.072**
	(0.030)	(0.025)	(0.038)	(0.032)	(0.032)
Family ties (weighted)	0.055*	0.069***	0.085**	0.074**	0.070**
	(0.031)	(0.024)	(0.035)	(0.030)	(0.032)
N	98,739	98,739	98,739	43,120	75,115
Panel B2: 18-64					
Family ties	0.055*	0.075***	0.084**	0.084**	0.079**
	(0.031)	(0.025)	(0.035)	(0.033)	(0.032)
Family ties (weighted)	0.056*	0.075***	0.088***	0.083***	0.077**
	(0.032)	(0.024)	(0.032)	(0.031)	(0.032)
N	86,252	86,252	86,252	35,003	67,859
Panel B3: 18-34					

Family ties	0.029	0.069**	0.081***	0.100***	0.097***
	(0.038)	(0.028)	(0.030)	(0.036)	(0.027)
Family ties (weighted)	0.030	0.070**	0.084***	0.101***	0.094***
	(0.039)	(0.028)	(0.029)	(0.033)	(0.028)
N	38,016	38,016	38,016	11,495	32,994

Table S6. Historical climate variability as alternative family ties measure.

Data drawn from 39 European countries with historical climate data.

Panel A	Strength of family ties	Strength of family ties (A & G, 2010)	Principal component variability	Temperature variability	Precipitation variability
Strength of family ties	1.0000				
Strength of family ties (A & G, 2010)	0.6302	1.0000			
Principal component variability	-0.5519	-0.5446	1.0000		
Temperature variability	-0.6981	-0.6290	0.9127	1.000	
Precipitation variability	-0.3091	-0.3649	0.9126	0.6658	1.0000

Panel B	Hand-washing frequency	Avoid crowds	Self-quarantine	Vaccine for self	Mandatory vaccination
Panel B1: All age					
Principal component variability	-0.099***	-0.060*	-0.090	-0.054*	-0.102
	(0.022)	(0.031)	(0.056)	(0.031)	(0.108)
N	59,629	59,629	59,629	31,453	36,590
Panel B2: 18-64					
Principal component variability	-0.101***	-0.059	-0.078	-0.036	-0.015
	(0.024)	(0.037)	(0.061)	(0.029)	(0.049)
N	50,141	50,141	50,141	24,758	32,231
Panel B3: 18-34					
Principal component variability	-0.089***	-0.044	-0.041	-0.026	-0.083
	(0.026)	(0.053)	(0.072)	(0.028)	(0.112)
N	19,590	19,590	19,590	7,549	14,507

Table S7. Regression results controlling for institutional collectivism social values and practices. Data drawn from 49 countries in the GLOBE study.

	Hand-washing frequency	Avoid crowds	Self-quarantine	Vaccine for self	Mandatory vaccination
Panel A1: All age					
Family ties	0.075**	0.067**	0.077**	0.076***	0.063
	(0.030)	(0.027)	(0.035)	(0.023)	(0.040)
Institutional Collectivism values	0.013	0.080**	0.029	0.142***	0.220***
	(0.031)	(0.036)	(0.044)	(0.046)	(0.041)
Institutional Collectivism practices	-0.015	0.008	-0.013	0.143***	0.182***
	(0.026)	(0.030)	(0.027)	(0.027)	(0.059)
Family ties (weighted)	0.077***	0.069***	0.078**	0.076***	0.062
	(0.029)	(0.026)	(0.033)	(0.023)	(0.039)
Institutional Collectivism values	0.016	0.083**	0.032	0.144***	0.222***
	(0.029)	(0.035)	(0.044)	(0.046)	(0.041)
Institutional Collectivism practices	-0.013	0.009	-0.012	0.144***	0.183***
	(0.025)	(0.029)	(0.027)	(0.028)	(0.059)
N	94,789	94,789	94,789	43,858	74,620
Panel A2: 18-64					
Family ties	0.075**	0.068**	0.080**	0.073***	0.069
	(0.032)	(0.027)	(0.032)	(0.023)	(0.043)
Institutional Collectivism values	0.017	0.088**	0.045	0.141***	0.223***
	(0.031)	(0.035)	(0.042)	(0.043)	(0.044)
Institutional Collectivism practices	-0.012	0.013	-0.003	0.141***	0.189***
	(0.026)	(0.029)	(0.024)	(0.027)	(0.061)
Family ties (weighted)	0.077**	0.071***	0.079**	0.072***	0.068
	(0.030)	(0.026)	(0.031)	(0.022)	(0.042)
Institutional Collectivism values	0.020	0.091***	0.048	0.143***	0.225***
	(0.030)	(0.034)	(0.042)	(0.043)	(0.043)
Institutional Collectivism practices	-0.010	0.015	-0.002	0.141***	0.190***
	(0.026)	(0.028)	(0.025)	(0.028)	(0.061)
N	80,969	80,969	80,969	34,474	67,302
Panel A3: 18-34					
Family ties	0.070**	0.081***	0.095***	0.086***	0.101**
	(0.032)	(0.025)	(0.030)	(0.026)	(0.045)

Institutional Collectivism values	0.006	0.089***	0.062	0.125***	0.208***
	(0.034)	(0.032)	(0.043)	(0.038)	(0.050)
Institutional Collectivism practices	-0.000	0.018	0.001	0.130***	0.180***
	(0.028)	(0.028)	(0.024)	(0.029)	(0.061)
Family ties (weighted)	0.071**	0.081***	0.092***	0.083***	0.099**
	(0.031)	(0.023)	(0.030)	(0.027)	(0.043)
Institutional Collectivism values	0.008	0.092***	0.064	0.127***	0.211***
	(0.034)	(0.032)	(0.043)	(0.038)	(0.049)
Institutional Collectivism practices	0.001	0.019	0.002	0.131***	0.181***
	(0.028)	(0.027)	(0.024)	(0.029)	(0.061)
N	33,253	33,253	33,253	10,337	31,400

Table S8. Regression results controlling for 7-day average country-level vaccination coverage 30 days prior to PsyCorona survey wave.

	Hand-washing frequency	Avoid crowds	Self-quarantine	Vaccine for self	Mandatory vaccination
Panel A1: All age					
Family ties	0.063**	0.075**	0.110***	0.065*	0.083
	(0.030)	(0.030)	(0.034)	(0.031)	(0.051)
Family ties (weighted)	0.063**	0.074**	0.104***	0.061**	0.076
	(0.028)	(0.030)	(0.033)	(0.028)	(0.049)
N	111,526	111,526	111,526	50,025	88,329
Panel A2: 18-64					
Family ties	0.063**	0.078**	0.114***	0.069**	0.090*
	(0.030)	(0.031)	(0.034)	(0.032)	(0.051)
Family ties (weighted)	0.063**	0.077**	0.107***	0.063**	0.082*
	(0.028)	(0.031)	(0.033)	(0.029)	(0.050)
N	96,547	96,547	96,547	39,934	80,372
Panel A3: 18-34					
Family ties	0.044	0.083***	0.119***	0.101***	0.119**
	(0.039)	(0.032)	(0.030)	(0.038)	(0.049)
Family ties (weighted)	0.044	0.080***	0.113***	0.091***	0.111**
	(0.036)	(0.031)	(0.029)	(0.034)	(0.047)
N	41,647	41,647	41,647	12,699	39,134

Table S9. Regression results with family ties constructed by the same three survey questions in Alesina and Giuliano (2010). Family ties measure was constructed by using WVS 2005-2009 due to the availability of survey questions used by these scholars. If a country did not participate in WVS 2005-2009, we used its most recent wave available prior to 2005 (up till 1989). And 76 countries were included in the analysis. In addition to rate how important family is, respondents are asked the following two questions: *Respect and love parents*, which asks whether (1) regardless of what the qualities and faults of one's parents are, one must always love and respect them, and (2) one does not have the duty to respect and love parents who have not earned it. *Parental duties*, which asks whether: (1) It is the parents' duty to do their best for their children, even at the expense of their own well-being, and (2) parents have a life on their own.

	Hand-washing frequency	Avoid crowds	Self-quarantine	Vaccine for self	Mandatory vaccination
Panel A1: All age					
Family ties	0.052**	0.058**	0.009	0.002	0.055
	(0.026)	(0.023)	(0.038)	(0.032)	(0.036)
Family ties (weighted)	0.053**	0.059**	0.007	0.003	0.059*
	(0.027)	(0.024)	(0.040)	(0.031)	(0.035)
N	109,575	109,575	109,575	49,137	75,115
Panel A2: 18-64					
Family ties	0.054**	0.062***	0.014	0.006	0.058
	(0.028)	(0.024)	(0.034)	(0.033)	(0.037)
Family ties (weighted)	0.056**	0.063***	0.012	0.006	0.062*
	(0.028)	(0.024)	(0.035)	(0.032)	(0.037)
N	94,629	94,629	94,629	39,049	67,859
Panel A3: 18-34					
Family ties	0.042	0.064**	0.035	0.012	0.071**
	(0.028)	(0.026)	(0.026)	(0.031)	(0.035)
Family ties (weighted)	0.043	0.066**	0.034	0.013	0.075**
	(0.029)	(0.026)	(0.027)	(0.031)	(0.035)
N	40,803	40,803	40,803	12,241	32,994

Table S10. Within-country standard deviation of family ties constructed by PCA and summation

Method	within group standard deviation
PCA	0.162
Sum	0.122

Table S11. Correlations among family values

	Family important	Parents proud	PCA	Sum
Family important	1.0000			
Parents proud	0.1443	1.0000		
PCA	0.1417	0.4553	1.0000	
Sum	0.1306	0.4923	0.9415	1.0000

Table S12. Comparison between individuals who continued and discontinued participation of PsyCorona survey. Respondents who have participated in at least one wave after baseline are in the “continued” subsample. Individuals answered “willingness for receive vaccine” question are in the “continued” subsample automatically.

	Discontinue			Continue			P-value
	Mean	SD	observations	Mean	SD	observations	
Panel A: All age							
Hand-washing frequency	6.262	0.005	44,768	6.44	0.007	16,631	0.000
Avoid crowds	6.365	0.005	44,768	6.603	0.006	16,631	0.000
Self-quarantine	5.813	0.007	44,768	5.904	0.012	16,631	0.000
Mandatory vaccination	5.264	0.009	44,813	5.310	0.015	16,631	0.006
Age	2.780	0.007	44,768	3.314	0.013	16,631	0.000
Female	0.592	0.002	44,768	0.662	0.004	16,631	0.000
Education	0.750	0.002	44,768	0.774	0.003	16,631	0.000
Unemployed	0.094	0.001	44,768	0.167	0.003	16,631	0.000
Financially strained	2.025	0.006	44,768	1.876	0.010	16,631	0.000
Panel B: 18-34							
Hand-washing frequency	6.200	0.008	22,654	6.409	0.012	6,233	0.000
Avoid crowds	6.325	0.007	22,654	6.607	0.010	6,233	0.000
Self-quarantine	5.867	0.010	22,654	6.082	0.017	6,233	0.000
Mandatory vaccination	5.326	0.012	22,673	5.396	0.023	6,233	0.006
Age	1.494	0.003	22,654	1.614	0.006	6,233	0.000
Female	0.621	0.003	22,654	0.740	0.006	6,233	0.000
Education	0.762	0.003	22,654	0.811	0.005	6,233	0.000
Unemployment	0.109	0.002	22,654	0.212	0.005	6,233	0.000
Financially strained	2.025	0.008	22,654	1.846	0.016	6,233	0.000

SI References

Alesina, A., & Giuliano, P. (2010). The power of the family. *Journal of Economic Growth*, *15*(2), 93-125. https://doi.org/10.1007/s10887-010-9052-z

Luterbacher, J., Dietrich, D., Xoplaki, E., Grosjean, M., & Wanner, H. (2004). European Seasonal and Annual Temperature Variability, Trends, and Extremes Since 1500. *Science*, *303*(5663), 1499-1503. https://doi.org/10.1126/science.1093877

Pauling, A., Luterbacher, J., Casty, C., & Wanner, H. (2006). Five hundred years of gridded high-resolution precipitation reconstructions over Europe and the connection to large-scale circulation. *Climate Dynamics*, *26*(4), 387-405. https://doi.org/10.1007/s00382-005-0090-8
